# Insights into the genomic architecture of a newly discovered endophytic *Fusarium* species belonging to the *Fusarium concolor* complex from India

**DOI:** 10.3389/fmicb.2023.1266620

**Published:** 2023-11-27

**Authors:** Shiwali Rana, Sanjay K. Singh

**Affiliations:** National Fungal Culture Collection of India, Biodiversity and Palaeobiology Group, MACS' Agharkar Research Institute, Pune, India

**Keywords:** asexual morph, novel taxon, *Fusarium*, genome mining, antiSMASH, secondary metabolites

## Abstract

In this study, a new species *Fusarium indicum* belonging to the *Fusarium concolor* species complex is established to accommodate an endophytic fungus isolated from *Bambusa* sp. and collected from Himachal Pradesh. The identity of this isolate was confirmed based on the asexual morphs, its cultural characteristics, and phylogenetic analyses. This isolate revealed out to be distinct by showing less similarity with described species in the genus *Fusarium* based on molecular sequence data, approximately 93.9% similarity based on translation elongation factor 1-alpha, and 94.2% similarity based on RNA polymerase II subunit. Furthermore, to increase knowledge about this novel species, whole-genome sequencing was carried out. The results displayed that *Fusarium indicum* NFCCI 5145 possesses a 40.2 Mb genome and 48.39% of GC content. Approximately 12,963 functional protein-coding genes were carefully predicted and annotated using different BLAST databases, such as Uniprot, Kyoto Encyclopedia of Genes and Genomes (KEGG), Gene Ontology (GO), Pathogen Host Interactions (PHI), Clusters of Orthologous Groups (COG), and Carbohydrate-Active enzymes (CAZy). The orthologous proteins were identified using OrthoFinder and used for the phylogenetic analysis. ANIb confirmed that the isolate is closely related to the *F. concolor* species complex. It is known that *Fusarium* strains can produce a wide range of bioactive secondary metabolites. Therefore, in-depth mining for biosynthetic gene clusters for secondary metabolite biosynthesis of *Fusarium indicum* NFCCI 5145 was investigated using Antibiotics and Secondary Metabolites Analysis Shell (AntiSMASH) annotation. AntiSMASH results displayed that this isolate possesses 45 secondary metabolites of biosynthetic gene clusters (BGCs). These findings significantly improved our understanding of the strain *Fusarium indicum* NFCCI 5145 and its possible applications in different sectors including industry for the secondary metabolites and enzymes it can produce.

## 1 Introduction

The first report on the genus *Fusarium* was described by Link in 1809 (Link, 1809). *Fusarium* species are present in almost any ecosystem globally (Leslie and Summerell, 2006). They have been reported from nearly all bioclimatic regions of the world, including tropical and temperate grasslands, shrublands, forests, harsh desert, and alpine environments, soils associated with plants, organic debris, and any part of plants from plants’ deepest roots to highest flowers (Leslie and Summerell, 2006). Therefore, *Fusarium* species can colonize multiple habitats in almost all ecosystems worldwide (Young et al., 1978; Nelson et al., 1994; Arney et al., 1997). *Fusarium* is one of the most economically significant fungi, mostly known as a pathogen, capable of infecting essential agricultural and horticultural crops worldwide (Booth, 1971; Leslie and Summerell, 2006). *Fusarium* species are mainly responsible for wilts, blights, root rots, and cankers (Ingle, 2017). Its species can also occur as saprophytes, endophytes, parasites, pathogens of plants, and pathogens or mutualists of animals (Torbati et al., 2021).

*Fusarium concolor* was first established on *Hordeum vulgare* from Montevideo, Uruguay (Reinking, 1934) and later was reported from various hosts, such as wheat, banana, koa tree, Guarana, *Hybanthus prunifolius*, plant debris, and soil (Chambers, 1972; Saadabi, 2006; Murad and Baiti, 2017; Almeida et al., 2021). It has also been known to cause keratitis and fusariosis (Al-Hatmi et al., 2016). The *Fusarium concolor* species complex currently consists of four species, namely, *F*. *anguioides*, *F. austroafricanum*, *F. bambusarum,* and *F. concolor*. The isolates of *F. anguioides* have been reported from bamboo, *Cordyline stricta,* and *Alocasia odora*; *Fusarium bambusarum* have been reported from bamboo (Nelson et al., 1995; Wang et al., 2022). *Fusarium austroafricanum* was isolated as an endophyte of Kikuyu grass associated with putative mycotoxicosis of cattle (Jacobs-Venter et al., 2018). In the present study, an endophytic isolate was isolated from *Bambusa* sp. and was established as a new species of the genus *Fusarium,* falling into the *Fusarium concolor* species complex based on morphology and phylogeny. Furthermore, whole-genome sequencing was targeted to get insights into the genomic architecture of this newly discovered *Fusarium* species.

## 2 Materials and methods

### 2.1 Collection, isolation, and morphological characterization of endophytic *Fusarium indicum*

Healthy leaves of *Bambusa* sp. collected from Panchrukhi, Palampur, District Kangra of Himachal Pradesh, India, on 4 October 2021 were placed in sterile polythene bags and transported carefully to the laboratory. The surface adherents were removed after thorough washing under tap water. Then, bigger leaves were chopped into small pieces and subjected to surface sterilization following a modified method by Dobranic et al. (1995). Concisely, bamboo leaves were first dipped in 70% ethanol for 5 s, followed by 4% sodium hypochlorite for 90 s, and later rinsed with sterile water for 10 s (four times). These surface sterilized leaves were cut into small pieces using a sterilized sharp blade and inoculated on potato dextrose agar (PDA) plates. These plates were kept at 25°C until any vegetative growth appeared from the inoculated tissues. Individual colonies arising from inoculated tissues were transferred to fresh PDA plates by hyphal tipping and allowed to grow (Bills and Polishook, 1992). Furthermore, pure culture was raised using a single spore isolation technique. Colony characteristics of this isolate were studied on PDA and synthetic nutrient agar (SNA). Methuen’s Handbook of Color was referred to recording the colors of the colonies on different agar media (Kornerup and Wanscher, 1978). Microscopic structures of the isolates were recorded from pure culture using staining *cum*-mounting medium, lactophenol cotton blue, under a Carl Zeiss Image Analyzer 2 (Germany) microscope. Measurements and photomicrographs of the fungal structures were recorded using Axiovision Rel 4.8 software and Digi-Cam attached with Carl Zeiss Image Analyzer 2 microscope. The holotype specimen is deposited and accessioned in Ajrekar Mycological Herbarium (AMH 10381), and ex-type pure culture is deposited and accessioned in the National Fungal Culture Collection of India (NFCCI 5145).

### 2.2 DNA extraction, PCR amplification, and DNA sequencing

Genomic DNA was extracted from pure colonies raised from single spore isolation on PDA Petri plates. After approximately 1 week of incubation, DNA extraction was performed by a simple, easy, and rapid DNA extraction protocol using FastPrep®24 tissue homogenizer (MP Biomedicals GmbH, Eschwege, Germany; Aamir et al., 2015). The amplification and sequencing of *tef-1α*, *rpb2,* and LSU gene regions were carried out. The primers involved in amplification and sequencing were EF-1 (5′ ATGGGTAAGGARGACAAGAC 3′) and EF-2 (5′ GGARGTACCAGTSATCATG 3′) for translation elongation factor 1-alpha (*tef1-α*; O’Donnell et al., 1998), LR-OR (5′ ACCCGCTGAACTTAAGC 3′; Vilgalys and Sun, 1994), LR-7 (5′ TACTACCACCAAGATCT 3′; Vilgalys and Hester, 1990) for 28S large subunit of the nrDNA (LSU), and fRPB2-5f (5′ GAYGAYMGWGATCAYTTYGG 3′) and fRPB2-7cR (5′ CCCATRGCTTGYTTRCCCAT 3′) for RNA polymerase second largest subunit (*rpb2*; Liu et al., 1999).

PCR was carried out in a 25 μL reaction using 12.5 μL 2x Invitrogen Platinum SuperFi PCR Mastermix, 2 μL template DNA (10–20 ng), 1.5 μL 10 pmol primer, 5 μL 5x GC enhancer, and H_2_O (Sterile Ultra-Pure Water, Sigma, St. Louis, MO, United States), with the total volume made to 25 μL. The conditions of the thermocycling involved those as follows: For *tef1-α* gene region, an initial denaturation of 5 min at 94°C, 30 cycles of 45 s at 94°C, 30 s at 57°C, and 1 min at 72°C followed by a final 7-min extension at 72°C; 5 min denaturation at 94°C, 35 cycles of 1 min at 94°C, 50 s at 52°C, and 1.2 min at 72°C, with a final 8 min extension at 72°C for LSU; and 5 min denaturation at 95°C, 35 cycles of 45 s at 95°C, 1 min at 52°C, and 1.5 min at 72°C, with a final 10 min extension at 72°C for *rpb2*.

The PCR amplicons were purified with a FavorPrep™ PCR purification kit as per the manufacturer’s instructions. Purified PCR products of all marker genes were checked on 1.2% agarose gel electrophoresis stained with 0.5 μg/mL ethidium bromide and were further subjected to a sequencing PCR using a BigDye®Terminator v3.1 Cycle Sequencing Kit, as per the manufacturer’s instructions. In brief, the sequencing PCR of 20 μL included 4 μL of 5× sequencing buffer, 2 μL of BigDye™ Terminator premix, 4 μL of primer (5 pmol), and 4 μL of the purified amplicon and H_2_O (Sterile Ultra-Pure Water, Sigma), with the volume made to 20 μL. Thermal cycling conditions consisted of an initial denaturing at 96°C for 3 min, followed by 30 cycles of 94°C for 10 s, 50°C for 40 s, and 60°C for 4 min. The BigDye® terminators and salts were removed using the BigDye Xterminator® Purification Kit (Thermo Fisher Scientific, Waltham, MA, United States) as per the manufacturer’s instructions. After performing cycle sequencing with BigDye™ terminators, 80 μL of SAM™ solution and 20 μL of XTerminator™ solution were added to each tube. The mixture was vortexed for 30 min and then centrifuged at 10,000 rpm for 30 s. The supernatant was transferred to a 96-well microplate, and the module was selected and run was set up. The sequence was elucidated on Applied Biosystems SeqStudio Genetic Analyzer (Applied Biosystems, Foster City, CA, United States). Sequences obtained were submitted in NCBI GenBank [accession numbers OM032811 (*tef-1α*), OM032812 (*rpb2*), and OM025235 (LSU)].

### 2.3 Phylogenetic analysis

To determine the phylogenetic status of this isolate, *tef-1α* and *rpb2* gene regions were used to compare the present isolate with already known authentic strains in the genus *Fusarium*. The sequences of the related authentic strains were retrieved from NCBI. A total of 83 isolates of the genus *Fusarium* were used in the phylogenetic analysis and were aligned along with the sequences of *Fusarium indicum* NFCCI 5145. *Geejayessia zealandica* CBS 111.93 and *Geejayessia cicatricum* CBS 125549 were selected as the outgroup taxa. The strains which were used in making phylogenetic tree, along with their accession numbers and other related details, are presented in Supplementary Table 1. Each gene region was aligned individually with MAFFT v. 6.864b (Katoh and Standley, 2013). The alignments were checked and adjusted manually using AliView (Larsson, 2014). Furthermore, alignments were concatenated and processed for the phylogenetic analyses. The best substitution model was figured using ModelFinder (Kalyaanamoorthy et al., 2017). Additionally, Windows version IQ-tree tool v.1.6.11 (Nguyen et al., 2015) was used to reconstruct the phylogenetic tree. The reliability of the tree branches was assessed and tested on the basis of 1,000 ultrafast bootstrap support replicates (UFBoot) and the SH-like approximate likelihood ratio test (SH-like aLRT) with 1,000 replicates. The constructed phylogenetic tree was visualized in FigTree v.1.4.4.

### 2.4 High molecular weight DNA extraction for whole-genome sequencing

In total, 100 mg of the fungal mass was crushed using a mortar-pestle in liquid N_2_. The powder was placed in a 2 mL sterile Eppendorf tube. Overall, 1 mL of pre-heated CTAB buffer [20 mM EDTA, 100 mM Tris HCl, and 1.4 M NaCl and CTAB 2%] was added along with 20 μL of β-mercaptoethanol and 1 mg of polyvinylpyrrolidone (PVP). The mixture was mixed properly and incubated at 65°C ± 2°C for at least 30 min. An equal amount of phenol:chloroform:isoamyl alcohol (25:24:1, v/v) was added and mixed well, and centrifuged at 10,000 rpm for 10 min. The upper aqueous layer was transferred to a fresh 1.5 mL Eppendorf tube and followed by the addition of an equal volume of chloroform:isoamyl alcohol (24:1, v/v) and mixed well and then centrifuged at 10,000 rpm for 10 min. The upper aqueous layer was transferred to a fresh 1.5 mL Eppendorf tube, and an equal volume of isopropanol was added and incubated under cold conditions at −20°C for 20 min. Furthermore, centrifugation was carried out at 10,000 rpm for 10 min at 4°C. The supernatant was removed, and the pellet was carefully washed using 500 μL of 70% ethanol. Again, centrifugation was carried out at 10,000 rpm for 5 min at 4°C. The supernatant was discarded, and the pellet was dried. The pellet was dissolved in 70 μL of 1× TE buffer. In total, 1 μL of RNase A solution (20 mg mL^−1^) was added and later incubated at 37°C for at least 30 min. The integrity was evaluated by 1% agarose gel electrophoresis, and purity was accessed by a NanoDrop™ 1,000 Spectrophotometer (Thermo Fisher Scientific).

### 2.5 Library preparation and sequencing

Library construction was done using the QIASeq FX DNA library preparation protocol (Cat#180475) as per the manufacturer’s instructions. In total, 50 ng of Qubit quantified DNA was enzymatically fragmented, end-repaired, and A-tailed in the one-tube reaction using the FX enzyme mix provided in the QIASeq FX DNA kit. The end-repaired and adenylated fragments were subjected to adapter ligation, whereby the index-incorporated Illumina adapter was ligated to generate sequencing libraries. These libraries were subjected to 6 cycles of Indexing PCR [initial denaturation at 98°C for 2 min and cycling (98°C for 20 s, 60°C for 30 s, and 72°C for 30 s) and final extension at 72°C for 1 min] to enrich the adapter-tagged fragments. Finally, for purification of the amplified libraries, JetSeq Beads were used (Bio-68031). Furthermore, quantification of the sequencing library was carried out by a Qubit fluorometer (Thermo Fisher Scientific, MA, United States). The sequencing of the libraries was performed on an Illumina NovaSeq 6000 sequencer (Illumina, San Diego, CA) for 150 bp paired-end chemistry, according to the manufacturer’s procedure.

### 2.6 Gene prediction and annotation

Libraries were paired-end sequenced using an Illumina NovaSeq 6000 sequencer. After sequencing, the paired-end raw data were kept in FASTQ format. Fastp (0.20.1) tool was used to remove low-quality reads, adapters, and polyG tails from FastQ files.^1^ To get the draft genome sequence, St. Petersburg genome assembler SPAdes version 3.11.1 was used for assembly.^2^ The genome diagram of *Fusarium indicum* NFCCI 5145 was constructed using Circos version 0.69-9 (Krzywinski et al., 2009). Augustus 3.4.0 tool was used for performing gene prediction.^3^ The gene function and metabolic pathway data available in the already existing databases were used to carry out the function annotation, and BLAST searches were performed against various databases such as NR (NCBI non-redundant protein sequences), Uniprot, COG (Cluster of Orthologous Groups of proteins), KEGG (Kyoto Encyclopedia of Genes and Genomes), CAZy (Carbohydrate-Active Enzymes Database; Lombard et al., 2014), and PHI (Pathogen Host Interactions Database; Urban et al., 2020). Secondary metabolite biosynthetic gene clusters of *Fusarium indicum* NFCCI 5145 were analyzed using antiSMASH fungal 6.1.1 (Blin et al., 2021). Tandem Repeats Finder Program (trf) version 4.09.1 was used to predict the number of repeats^4^ (Benson, 1999).

### 2.7 Comparative genomics and phylogenetic analysis

Orthologous proteins were identified using OrthoFinder version 2.5.4 (Emms and Kelly, 2019), and the results were used to build a species tree, visualized using FigTree. *Fusarium* species used for the study of the orthologous proteins and phylogenetic tree construction are presented in Table 1. The analysis of average nucleotide identity (ANI) was performed using the pyani script and ANIb as an algorithm for the alignment (Pritchard et al., 2016). *Fusarium* species used in the analysis of ANI are presented in Table 2.

**Table 1:** *Fusarium* species used for the comparison of orthologous proteins with *Fusarium indicum* NFCCI 5145 and its phylogeny.

Sr. No.	*Fusarium* spp.	Collection and strain no.	Accession number
1	*Fusarium concolor*	NRRL 13459	GCA_013184415.1
2	*Fusarium austroafricanum*	NRRL 53441	GCA_012932025.1
3	*Fusarium redolens*	NRRL 28421	GCA_019843785.1
4	*Fusarium hostae*	NRRL 29888	GCA_013184365.1
5	*Fusarium oxysporum*	Fo47	GCA_013085055.1
6	*Fusarium oxysporum*	NRRL 32931	GCA_000271745.2
7	*Fusarium veterinarium*	F5_8S_1A_F	GCA_019191175.1
8	*Fusarium foetens*	NRRL 38302	GCA_013623845.1
9	*Fusarium nisikadoi*	NRRL 25179	GCA_013623555.1
10	*Fusarium miscanthi*	NRRL 26231	GCA_014898875.1
11	*Fusarium lyarnte*	NRRL 54252	GCA_014898885.1
12	*Fusarium commune*	JCM 11502	GCA_001599515.1
13	*Fusarium commune*	NRRL 28387	GCA_013618355.1
14	*Fusarium newnesense*	NRRL 66241	GCA_013184375.1
15	*Fusarium tupiense*	NRRL 53984	GCA_013364945.1
16	*Fusarium xylarioides*	NRRL 25486	GCA_013623735.1
17	*Fusarium xylarioides*	K1	GCA_013183765.1
18	*Fusarium udum*	NRRL 25194	GCA_013186905.1
19	*Fusarium thapsinum*	NRRL 22049	GCA_013186935.1
20	*Fusarium xyrophilum*	NRRL 62721	GCA_008711595.1
21	*Fusarium xyrophilum*	NRRL 66890	GCA_008711575.1
22	*Fusarium xyrophilum*	NRRL 62710	GCA_008711615.1
23	*Fusarium verticillioides*	NRRL 20984	GCA_013759275.1
24	*Fusarium tjaetaba*	NRRL 66243	GCA_013396195.1
25	*Fusarium beomiforme*	NRRL 25174	GCA_002980475.2
26	*Fusarium burgessii*	NRRL 66654	GCA_002980515.1
27	*Fusarium algeriense*	NRRL 66647	GCA_002982055.1
28	*Fusarium algeriense*	NRRL 66648	GCA_002982035.1
29	*Fusarium avenaceum*	MPI-SDFR-AT-0044	GCA_020744115.1
30	*Fusarium tricinctum*	NRRL 25481	GCA_012977725.1
31	*Fusarium heterosporum*	NRRL 20693	GCA_013396295.1
32	*Fusarium buharicum*	NRRL 13371	GCA_014822075.1
33	*Fusarium sublunatum*	NRRL 13384	GCA_013623665.1
34	*Fusarium sarcochroum*	NRRL 20472	GCA_013266185.1
35	*Fusarium continuum*	NRRL 66286	GCA_013184455.1
36	*Fusarium torreyae*	NRRL 54149	GCA_014824505.1
37	*Fusarium zanthoxyli*	NRRL 66285	GCA_013623745.1
38	*Fusarium camptoceras*	NRRL 13381	GCA_004367475.1
39	*Fusarium scirpi*	NRRL 66328	GCA_004367495.1
40	*Fusarium tanahbumbuense*	NRRL 66471	GCA_012977735.1
41	*Fusarium ussurianum*	CBS 123752	GCA_017656685.1
42	*Fusarium vorosii*	CBS 119178	GCA_017656575.1
43	*Fusarium subtropicale*	NRRL 66764	GCA_003670145.1
44	*Fusarium sibiricum*	NRRL 53430	GCA_014898995.1
45	*Fusarium venenatum*	A3/5	GCF_900007375.1
46	*Fusarium venenatum*	NRRL 66329	GCA_013623635.1
47	*Fusarium transvaalense*	NRRL 31008	GCA_013623685.1
48	*Fusarium chlamydosporum*	NRRL 13444	GCA_014898915.1
49	*Fusarium nelsonii*	NRRL 13338	GCA_014898925.1
50	*Fusarium aywerte*	NRRL 25410	GCA_013186375.1
51	*Neocosmospora solani*	FSSC 5 MPI-SDFR-AT-0091	GCF_020744495.1
52	*Geejayessia zealandicum*	NRRL 22465	GCA_013266195.1
53	*Geejayessia cicatricum*	NRRL 54954	GCA_021730345.1

**Table 2:** Details of *Fusarium* species used for the calculation of genome-scale average nucleotide identity with *Fusarium indicum* NFCCI 5145.

Sr. no.	*Fusarium* spp.	Collection and strain no.	Accession number
1	*Fusarium concolor*	NRRL 13459	GCA_013184415.1
2	*Fusarium austroafricanum*	NRRL 53441	GCA_012932025.1
3	*Fusarium redolens*	NRRL 28421	GCA_019843785.1
4	*Fusarium hostae*	NRRL 29888	GCA_013184365.1
5	*Fusarium oxysporum*	Fo47	GCA_013085055.1
6	*Fusarium oxysporum*	NRRL 32931	GCA_000271745.2
7	*Fusarium veterinarium*	F5_8S_1A_F	GCA_019191175.1
8	*Fusarium foetens*	NRRL 38302	GCA_013623845.1
9	*Fusarium nisikadoi*	NRRL 25179	GCA_013623555.1
10	*Fusarium miscanthi*	NRRL 26231	GCA_014898875.1
11	*Fusarium lyarnte*	NRRL 54252	GCA_014898885.1
12	*Fusarium commune*	JCM 11502	GCA_001599515.1
13	*Fusarium commune*	NRRL 28387	GCA_013618355.1
14	*Fusarium newnesense*	NRRL 66241	GCA_013184375.1
15	*Fusarium tupiense*	NRRL 53984	GCA_013364945.1
16	*Fusarium xylarioides*	NRRL 25486	GCA_013623735.1
17	*Fusarium xylarioides*	K1	GCA_013183765.1
18	*Fusarium udum*	NRRL 25194	GCA_013186905.1
19	*Fusarium thapsinum*	NRRL 22049	GCA_013186935.1
20	*Fusarium xyrophilum*	NRRL 62721	GCA_008711595.1
21	*Fusarium xyrophilum*	NRRL 66890	GCA_008711575.1
22	*Fusarium xyrophilum*	NRRL 62710	GCA_008711615.1
23	*Fusarium verticillioides*	NRRL 20984	GCA_013759275.1
24	*Fusarium tjaetaba*	NRRL 66243	GCA_013396195.1
25	*Fusarium beomiforme*	NRRL 25174	GCA_002980475.2
26	*Fusarium burgessii*	NRRL 66654	GCA_002980515.1
27	*Fusarium algeriense*	NRRL 66647	GCA_002982055.1
28	*Fusarium algeriense*	NRRL 66648	GCA_002982035.1
29	*Fusarium avenaceum*	MPI-SDFR-AT-0044	GCA_020744115.1
30	*Fusarium tricinctum*	NRRL 25481	GCA_012977725.1
31	*Fusarium heterosporum*	NRRL 20693	GCA_013396295.1
32	*Fusarium buharicum*	NRRL 13371	GCA_014822075.1
33	*Fusarium sublunatum*	NRRL 13384	GCA_013623665.1
34	*Fusarium sarcochroum*	NRRL 20472	GCA_013266185.1
35	*Fusarium continuum*	NRRL 66286	GCA_013184455.1
36	*Fusarium torreyae*	NRRL 54149	GCA_014824505.1
37	*Fusarium zanthoxyli*	NRRL 66285	GCA_013623745.1
38	*Fusarium camptoceras*	NRRL 13381	GCA_004367475.1
39	*Fusarium scirpi*	NRRL 66328	GCA_004367495.1
40	*Fusarium tanahbumbuense*	NRRL 66471	GCA_012977735.1
41	*Fusarium ussurianum*	CBS 123752	GCA_017656685.1
42	*Fusarium vorosii*	CBS 119178	GCA_017656575.1
43	*Fusarium subtropicale*	NRRL 66764	GCA_003670145.1
44	*Fusarium sibiricum*	NRRL 53430	GCA_014898995.1
45	*Fusarium venenatum*	A3/5	GCF_900007375.1
46	*Fusarium venenatum*	NRRL 66329	GCA_013623635.1
47	*Fusarium transvaalense*	NRRL 31008	GCA_013623685.1
48	*Fusarium chlamydosporum*	NRRL 13444	GCA_014898915.1
49	*Fusarium nelsonii*	NRRL 13338	GCA_014898925.1
50	*Fusarium aywerte*	NRRL 25410	GCA_013186375.1

## 3 Results

### 3.1 Taxonomy of *Fusarium indicum*

**Figure 1:**
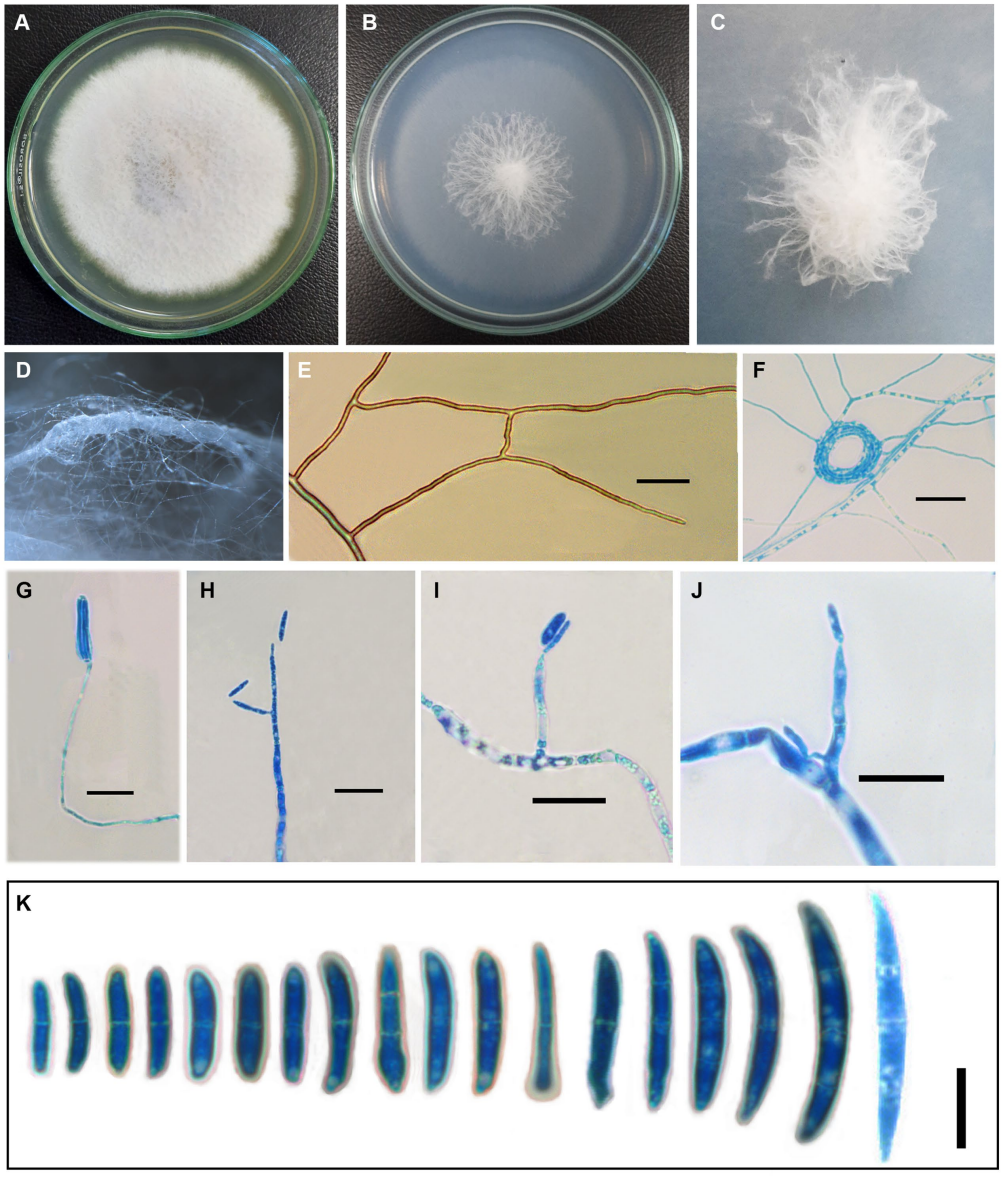
*Fusarium indicum* (NFCCI 5145); colonies of *Fusarium indicum* on **(A)** PDA; **(B)** SNA; **(C)** Stereoscopic view of colony growing on SNA; **(D)** Stereoscopic view of bundles of hyphae growing on SNA; **(E)** Hyphae showing anastomoses; **(F)** Coiled hyphae; **(G–J)** Phialides bearing microconidia; **(K)** Micro and macroconidia. —Scale bars: **E–J** = 20 μm, **K** = 10 μm.

**Figure 2:**
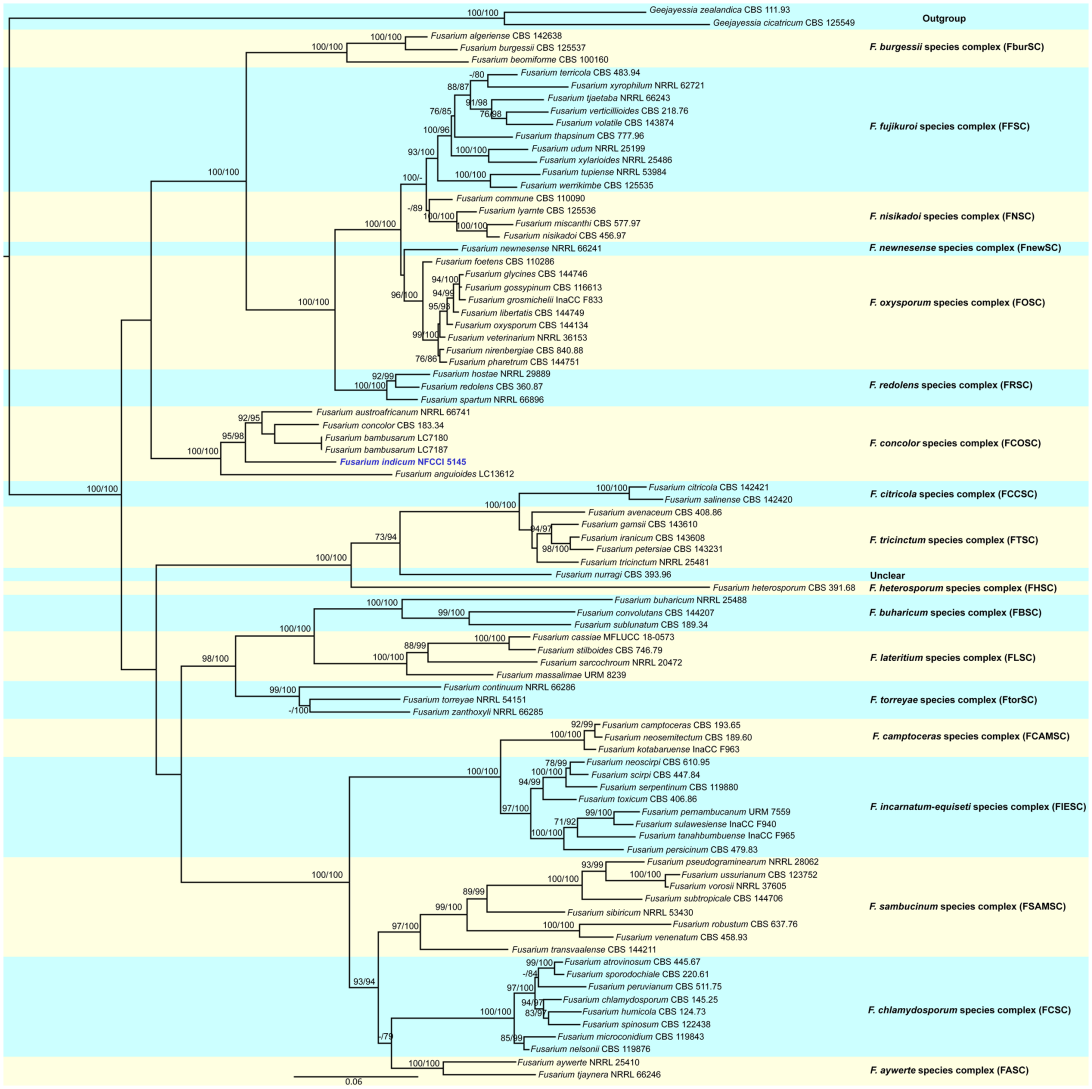
Molecular phylogenetic analysis of new species of *Fusarium indicum* (NFCCI 5145) based on maximum-likelihood (ML) method using both *tef-1α* and *rpb2* sequence data. New species *Fusarium indicum* (NFCCI 5145) is shown in blue. Statistical support values are shown next to each node, UFBS values and SH-aLRT obtained from 1,000 replicates using IQ-TREE and the TIM2e + I + G4 model.

*Fusarium indicum* S. Rana and S.K. Singh, sp. nov. Figures 1A–K, 2.

MycoBank Number: MB 847911

Holotype: AMH 10381

Etymology: Named after the country of isolation “India.”

Host/distribution: As endophyte from *Bambusa* sp. collected from Panchrukhi, Himachal Pradesh, India.

Original description: *Asexual morph Mycelium* appeared in parallel bundles of hyphae, simple to branched, septate, smooth-walled, hyaline, thin to thick, showing anastomoses, 1.0–6.5 μm wide (x̄ = 3.58 μm, *n* = 10). *Conidiophores* produced from superficial lateral hyphae, simple to branched, gradually decreasing to the length, septate, sometimes rudimentary protruding from superficial hyphae 24.67–118 × 1.35–4.20 μm (x̄ = 59.97 × 2.9 μm, *n* = 10). *Conidiogenous cells* are phialidic. *Phialides* produced directly from superficial hyphae, cylindrical gradually decreasing toward the length, ampuliform, monophialides to polyphialides, sometimes reduced to conidiogenous cell 4.5–43.70 × 2.25–4.90 μm (x̄ = 24.75 × 3.51 μm, *n* = 10). *Microconidia* rarely produced, hyaline, smooth-walled, fusoid to cylindrical, straight to curved, 0–1 septate, 9.5–19 × 2.15–4.5 μm (x̄ = 14.18 × 3.15 μm, *n* = 30). *Macroconidia* straight to curved, guttulate, short-beaked, 0–3 septate, foot cell prominent, 12.85–46.5 × 2.57–7.5 μm (x̄ = 23.04 × 3.73 μm, *n* = 30). *Chlamydospores* are rare, terminal to intercalary, sub-globose to globose, or solitary or in groups of 2–3.

Cultural characteristics: —Colonies growing on PDA reached 39 mm after 4 days at 25°C; yellowish white (1A2) to white (1A1), reverse pale yellow (3A3) after 1 week. In the colony, aerial mycelium is produced, colonies lacking distinct odor, colonies slightly raised, cottony, margin smooth and entire. Colonies growing on SNA reached 33 mm after 4 days of incubation at 25°C; grayish white (1B1) to white (1A1) after 1 week. Colony cottony, raised from the center, and flat toward the periphery with entire smooth margins.

*Sexual morph*: Not observed.

Known distribution: Panchrukhi, Himachal Pradesh, India.

Material examined: INDIA, Himachal Pradesh, Panchrukhi (32°03′01.1″N 76°34′01.8″E), from leaves of *Bambusa* sp., *S. rana*, 4 October 2021, AMH 10381(holotype), deposited in Ajrekar Mycological Herbarium (AMH) of India, ex-type culture is deposited in National Fungal Culture Collection of India (NFCCI 5145).

GenBank numbers: OM032811 (*tef-1α*), OM032812 (*rpb2*), and OM025235 (LSU).

*Notes*: The present species is somewhat similar to *F. concolor* morphologically (Reinking, 1934; Booth, 1971) in having pale pigmentation and marked heel on the foot cell of macroconidia. It is different from other taxa in the complex. The colonies of *F*. *anguioides* are carmine to ochre aerial mycelia, having red to brick tinges of pigmentation; *Fusarium austroafricanum* produces white to reddish white (7A2) colonies on PDA and reverse is light orange (6A4), while *F. indicum* produces white to yellowish white colonies on PDA. *F*. *concolor* differs from *F. indicum* in having smaller phialides compared with that of *F. indicum* and sometimes reduced to conidiogenous cell; aerial phialides monophialidic in *F*. *bambusarum* and polyphialidic in *F. concolor* vs. monophialidic to polyphialidic *F. indicum*. Macroconidia in *F. concolor* are usually 3–5 septate and occasionally 6–7 septate, *F. austroafricanum* macroconidia are 0–11 septate but mostly 3–8-septate, *F*. *anguioides* macroconidia 4–7 septate; *F*. *bambusarum* macroconidia 3–6 septate; however, *F. indicum* macroconidia are 0–3 septate (Booth, 1971; Jacobs-Venter et al., 2018; Yli-Mattila et al., 2018; Wang et al., 2022).

*Fusarium concolor* species are reported predominantly from soil; *F*. *anguioides* species have been reported from bamboo, *Cordyline stricta* and *Alocasia odora*; *Fusarium austroafricanum* was isolated as endophyte from *Pennisetum clandestinum* (kikuyu grass); *Fusarium bambusarum* species have been reported from bamboo (Booth, 1971; Nelson et al., 1995; Jacobs-Venter et al., 2018; Wang et al., 2022). Though *F*. *anguioides, F. bambusarum,* and *F. indicum* share the same host, they are quite distinct morphologically as well as based on the molecular data.

On the basis of MegaBLAST algorithm search on NCBI blastn^5^ for *Fusarium indicum* NFCCI 5145, the closest hit using *tef-1α* gene sequence was found to be *Fusarium concolor* (NRRL 13994, type) showing 92.59% (638 out of 689 bp) identity and having 15 gaps (2.17%) and with *Fusarium austroafricanum* (NRRL 66741, type) showing 93.48% (617 out of 660 bp) identity and having 4 gaps (0.6%); based on *rpb2,* closest hits were *Fusarium concolor* (NRRL 13994, type) showing 94.21% (766 out of 813 bp) identity and having 2 gaps (0.2%) and with *Fusarium austroafricanum* (NRRL 53441) showing 93.74% (644 out of 687 bp) identity and having 4 gaps (0.58%).

Additionally, the *tef-1α* gene sequence of *Fusarium indicum* NFCCI 5145 showed 93.54% (623 out of 666 bp) identity and had 6 gaps when compared with *Fusarium bambusarum* LC7180, and with *Fusarium anguioides* LC13612 showed 89.90% (595 out of 667 bp) identity and 10 gaps (1%; 10 out of 667 bp). Additionally, the *rpb2* gene sequence of *Fusarium indicum* NFCCI 5145 showed 93.63% (735 out of 785 bp) identity when compared with *Fusarium bambusarum* LC7180 and with *Fusarium anguioides* LC13612 showed 91.84% (721 out of 785 bp) identity.

### 3.2 Phylogenetic analysis

The sequence alignments of both *tef-1α* and *rpb2* were used to confirm the identity of this isolate. The concatenated file had sequence data of 86 taxa (Supplementary Table 1). Alignment contained 1,671 columns, 777 parsimony-informative, 1,061 distinct patterns, 113 singleton sites, and 781 constant sites. TIM2e + I + G4 was considered to be the best model and was selected based on the Bayesian Information Criterion (BIC). The phylogenetic tree was generated using the maximum likelihood method based on the above-mentioned model. The log-likelihood of the consensus tree was −24373.628. Rate parameters were A–C: 1.95361, A–G: 4.81458, A–T: 1.95361, C–G: 1.00000, C–T: 9.32964, and G–T: 1.00000; base frequencies were A: 0.250, C: 0.250, G: 0.250, and T: 0.250, and the proportion of invariable sites was 0.387 and gamma shape alpha parameter was 1.138. Combined phylogenetic analysis using *tef-1α* and *rpb2* nested this isolate, *Fusarium indicum*, in a distinct and unique clade in the *Fusarium concolor* species complex. The clade was well supported with strong SH-like aLRT and ultrafast bootstrap (UFBoot; Figure 2).

### 3.3 Genome sequencing and assembly of *Fusarium indicum* NFCCI 5145

**Figure 3:**
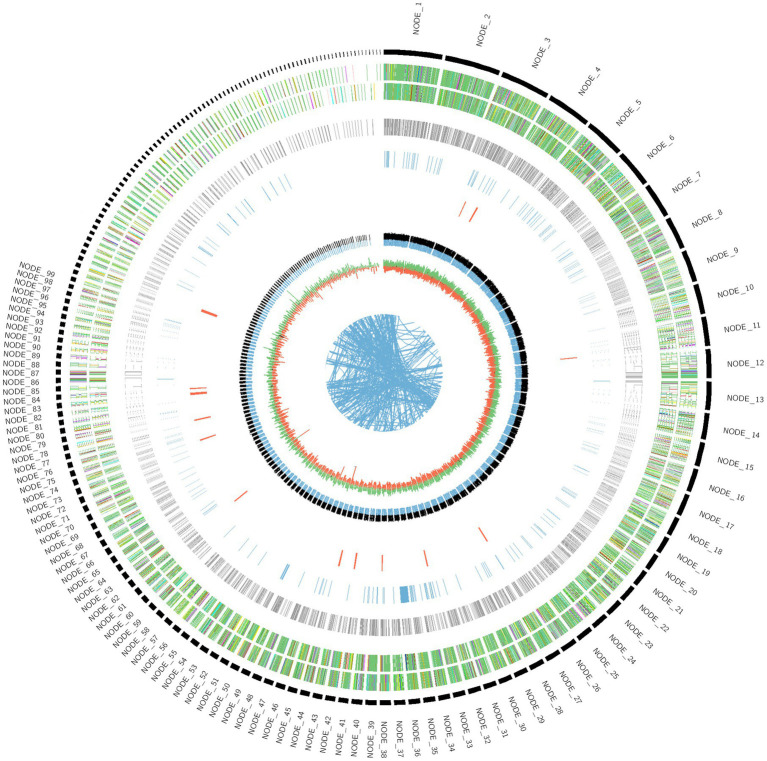
Genome diagram of strain *Fusarium indicum* NFCCI 5154.

The genome sequence of *Fusarium indicum* NFCCI 5145 was assembled and deposited in the NCBI GenBank database (SRA accession No. PRJNA993417; BioProject PRJNA993417; BioSample SAMN36633317; accession number JAWDHB000000000). The genome diagram of *Fusarium indicum* NFCCI 5145 shows that there are nine circles in the circle diagram (Figure 3), which are as follows from inside to outside: the first blue line shows in-paralog regions; the second circle shows the GC skew, with the green part showing a positive GC SKEW and the orange part showing a negative GC SKEW; the third circle shows the GC content (%; negative gene; positive gene); the fourth circle shows secondary metabolites; the fifth circle shows ncRNA; the sixth circle shows repeats; the seventh circle and the eighth circle display CDS annotation information. The seventh circle indicates that CDS is in a positive chain, and the eighth circle indicates that CDS is in a negative chain. The outer rim shows the contigs.

The whole genome size of *Fusarium indicum* NFCCI 5145 was 40.2 Mb. This consisted of 513 scaffolds with an N50 of 0.46 Mb and 48.39% of GC content. A total of 7,884,552 raw reads and 7,847,766 clean and high-quality reads (99.53%) were generated in the Illumina sequencing. Before filtering, total reads were 7.884552 M, total bases were 1.182683 G, Q20 bases were 1.162804 G (98.319170%), Q30 bases were 1.121368 G (94.815622%), and GC content was 48.005470%. After filtering, total reads were 7.847766 M, total bases were 1.176427 G, Q20 bases were 1.158196 G (98.450277%), Q30 bases were 1.117366 G (94.979678%), and GC content was 48.000585%. Reads that passed the filters were 7.847766 M (99.533442%), reads with low quality were 33.424000 K (0.423918%), reads with too many N were 2.328000 K (0.029526%), and reads which were too short were 1.034000 K (0.013114%). The total number of scaffolds was 513, the total number of bases (bp) were 40,226,774 (~40.2 Mb), the minimum scaffold length (bp) was 300, the maximum scaffold length (bp) was 1,668,425, scaffolds ≥ 200 kbp were 513, scaffolds ≥ 500 kbp were 347, scaffolds ≥ 1 kbp were 246, scaffolds ≥ 10 kbp were 169, scaffolds ≥ 1 Mbp were 6, and N50 value was 461,173. The Augustus prediction method was used to predict the encoding gene. In total, 12,724 protein-coding genes were predicted using highly annotated databases such as Uniprot. Gene total length (bp) was 6,336,400, gene average length (bp) was 498, maximum gene length (bp) was 8,599, and minimum gene length (bp) was 24. Simultaneously, the Tandem Repeats Finder Program (trf) was found to predict the number of repeats, which were found to be 3,232.

### 3.4 Genome sequence annotation of *Fusarium indicum* NFCCI 5145 using KEGG, COG, and GO

For the prediction of the protein sequences, 12,963 non-redundant genes of *Fusarium indicum* NFCCI 5145 were subjected to a similarity search on the basis of various public databases. Many genes were mapped using the Uniprot database (12,638 genes/97.49%), Clusters of Orthologous Groups (COG; 6,509 genes/50.21%), and Kyoto Encyclopedia of Genes and Genomes (KEGG; 9,155 genes/70.62%).

**Figure 4:**
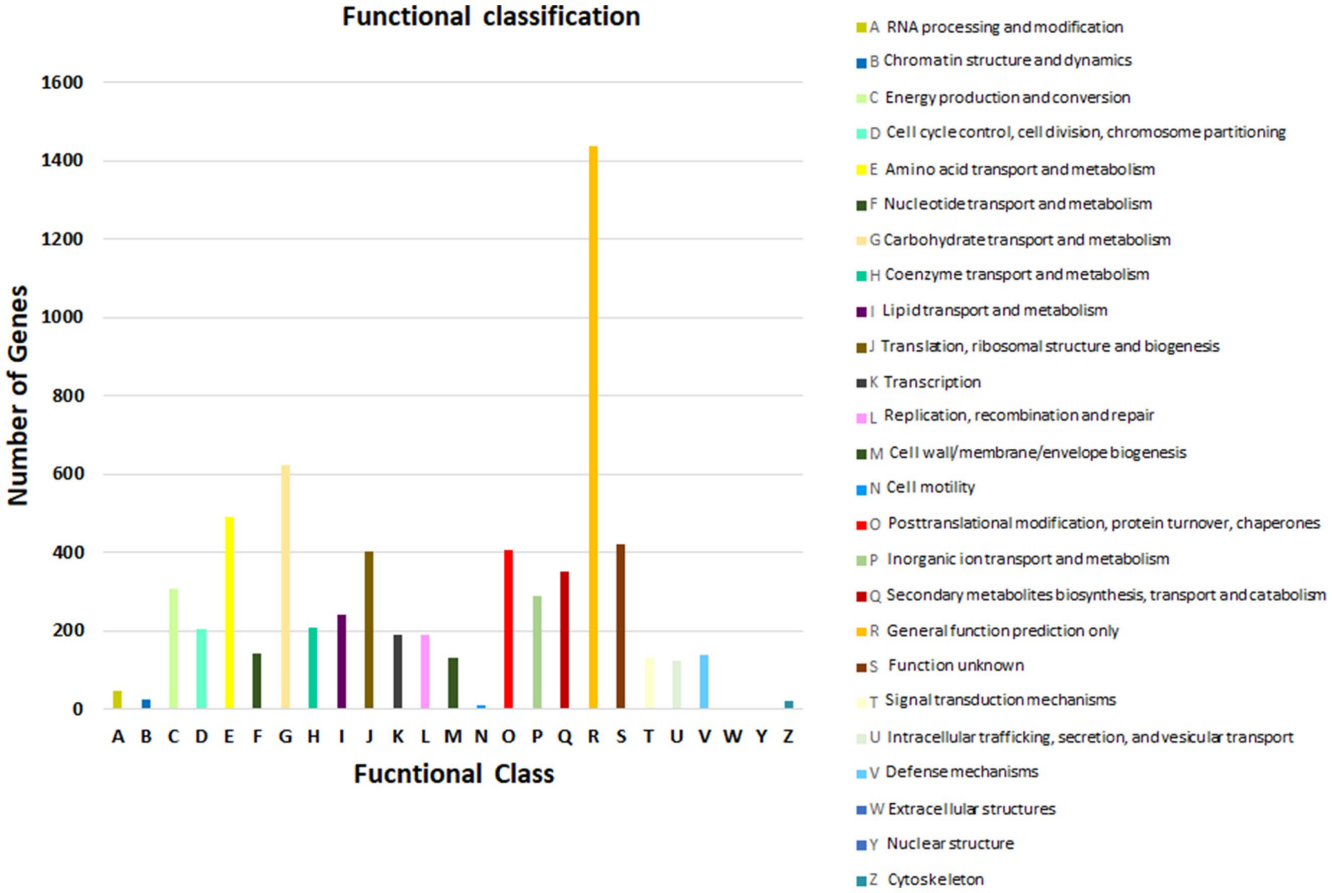
Functional annotation of *Fusarium indicum* NFCCI 5145 genes encoding for proteins using Clusters of Orthologous Genes (COG) database.

As per COG database, “General function prediction only” was related with many genes (1436), followed by “Carbohydrate transport and metabolism (624),” “Amino acid transport and metabolism (489),” “Function unknown (422),” “Post Translational modification, protein turnover, chaperones (405),” and “Translation, ribosomal structure and biogenesis (401)” (Figure 4; Natale et al., 2000). These results depict that *Fusarium indicum* NFCCI 5145 possesses a varied and enriched array of functions for carbohydrates and amino acid metabolism that may result in better energy conversion efficiency.

**Figure 5:**
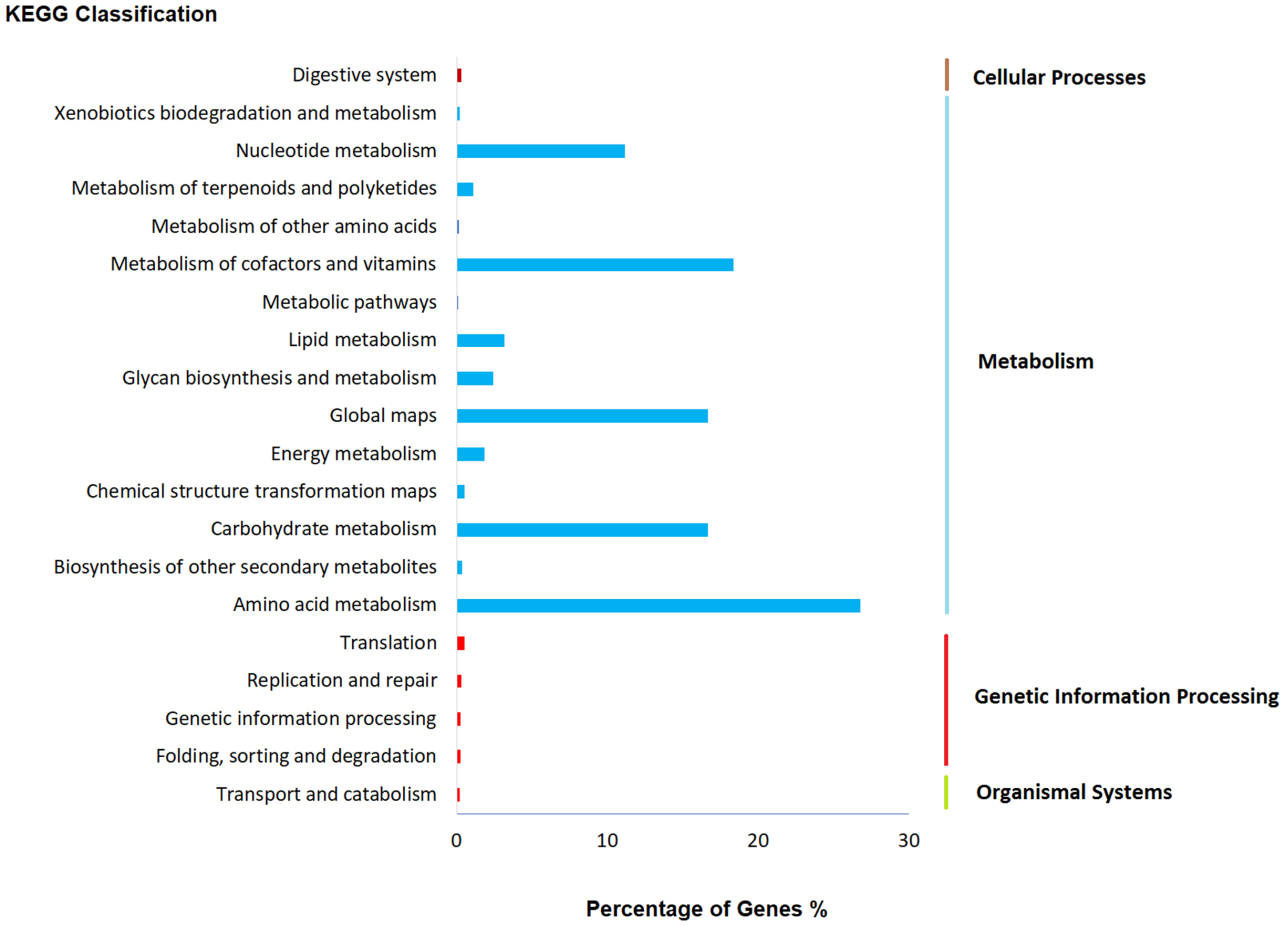
Functional annotation of *Fusarium indicum* NFCCI 5145 genes encoding for proteins using Kyoto Encyclopedia of Genes and Genomes (KEGG) analysis.

The findings from KEGG functional classification suggest that the predicted proteins fell under various categories, such as amino acid metabolism (2451), metabolism of cofactors and vitamins (1682), global map (1526), and carbohydrate metabolism (1525; Figure 5; Yamada et al., 2021). The results indicate that there is a varied and enriched array of various metabolic functions present that probably will provide higher secondary metabolism efficacy.

**Figure 6:**
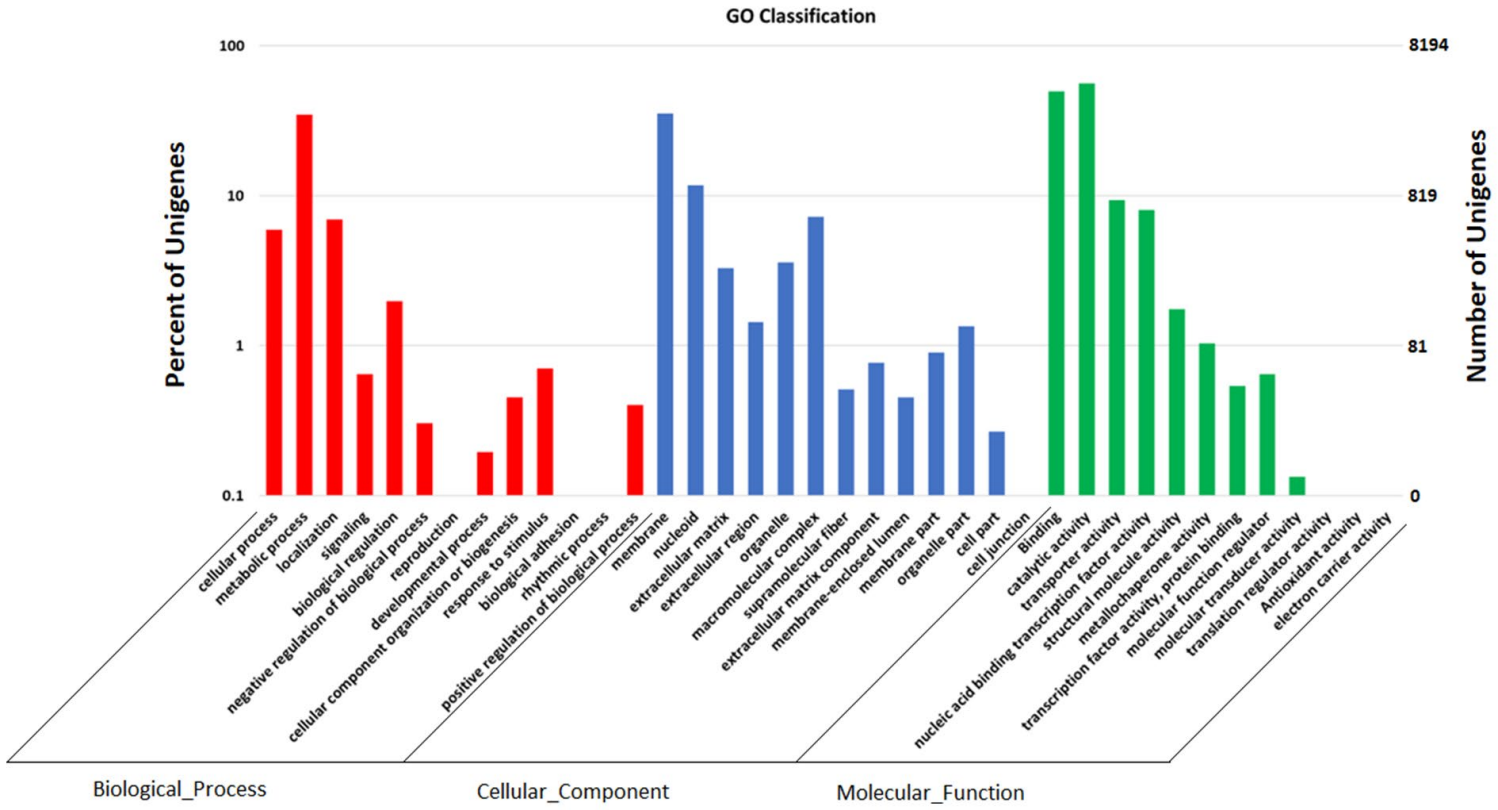
Functional annotation of *Fusarium indicum* NFCCI 5145 genes encoding for proteins using Gene Ontology (GO) analysis.

GO annotation depicts varied genes possessed by *Fusarium indicum,* which may be involved in biological processes, cellular components, and molecular functions (Figure 6; Huntley et al., 2014).

### 3.5 Genome sequence annotation of *Fusarium indicum* NFCCI 5145 for carbohydrate genes

**Figure 7:**
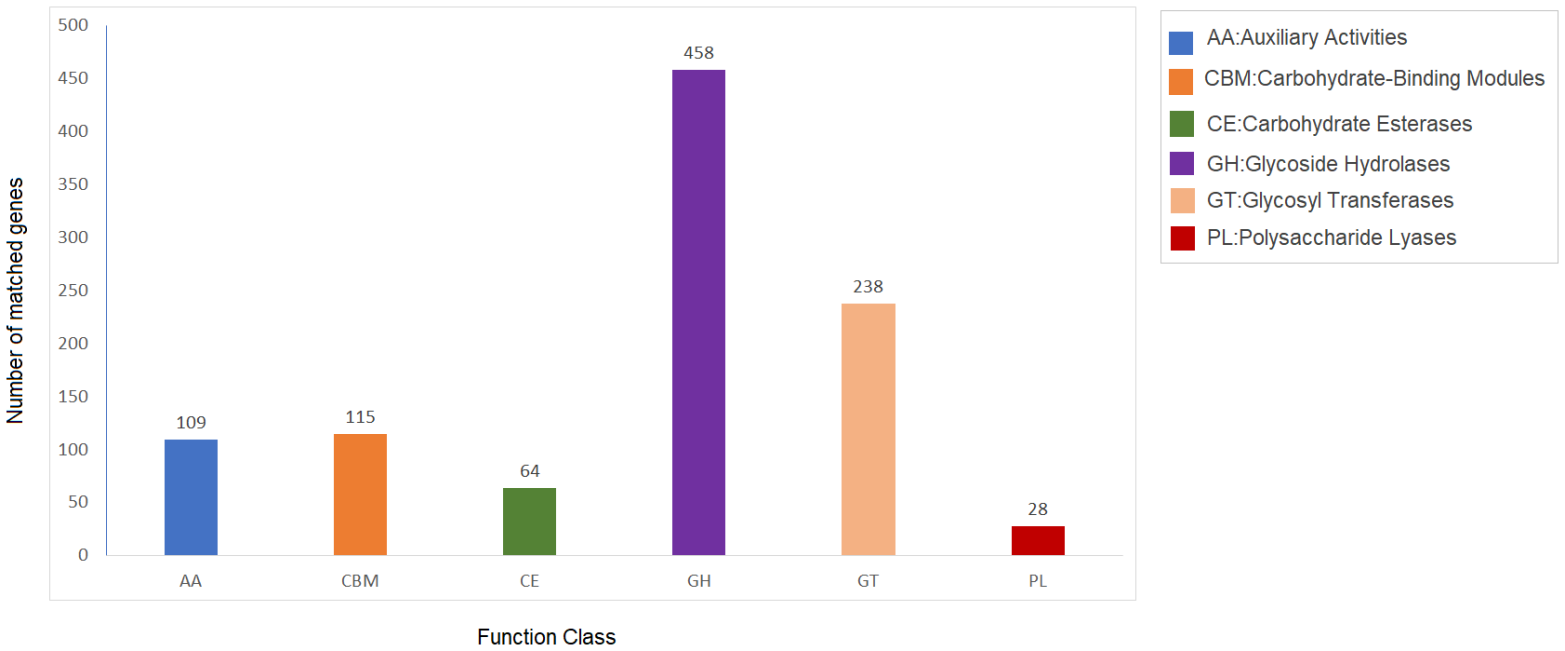
Carbohydrate-active enzymes (CAZy) functional classification and corresponding genes present in the genome of *Fusarium indicum* NFCCI 5145.

Carbohydrate-active enzymes (CAZymes) are classes of enzymes that catalyze the breakdown and assembly of glycoconjugates as well as glycans (oligosaccharides and polysaccharides; Garron and Henrissat, 2019). These enzymes play an important role in fungal metabolism as they are accountable for carbohydrate degradation and modification as well as biosynthesis (Liu et al., 2022). The CAZy is a specialized database for carbohydrate enzymes that have the capability to create, modify, and degrade glycosidic bonds (Liu et al., 2022). The analysis showed 1,012 genes that encode for carbohydrate-active enzymes (CAZy) dispersed in the genome of *Fusarium indicum* NFCCI 5145. These include 109 auxiliary activities (AAs), 115 carbohydrate-binding modules (CBMs), 64 carbohydrate esterases (CEs), 458 glycoside hydrolases (GHs), 238 glycosyltransferases (GTs), and 28 polysaccharide lyases (PLs; Figure 7). AAs mainly included AA1-16 16 families. CBMs distributed mainly across 23 families, including CBM1–2, CBM5–6, CBM9, CBM12–14, CBM18, CBM20–21, CBM24, CBM32, CBM35, CBM38, CBM41–43, CBM48, CBM50, CBM63, CBM67, and CBM87. Carbohydrate esterases were classified as CE1–5, CE8–9 CE12, CE14, and CE16 across 10 families. Glycoside hydrolases were distributed across 76 families, including GH1–3, GH5, GH7, GH9–13, GH15–18, GH20, GH23–33, GH35–39, GH43, GH47, GH49, GH51, GH53, GH55, GH62–65, GH67, GH71–79, GH81, GH86, GH88, GH92–95, GH103, GH105, GH114–115, GH123, GH125, GH128, GH130–132, GH135–136, GH139, GH141, GH146, GH152, GH154, and GH162. GTs contained 43 families, including GT1–4, GT8, GT10, GT11, GT15, GT17–18, GT20–24, GT28, GT30–35, GT39, GT41, GT43, GT47–48, GT50–51, GT57–59, GT61–62, GT64, GT66, GT69, GT71, GT76, GT90, GT106, GT107, and GT109. PLs are mainly distributed in eight families, including PL1, PL4, PL3, PL9, PL22, PL26, PL35 and PL42. These findings show that *Fusarium indicum* NFCCI 5145 can prove to show great ability to make and break complex carbohydrates making it capable of capturing more energy, thus possibly having great industrial applicability.

### 3.6 Genome sequence annotation of *Fusarium indicum* NFCCI 5145 for pathogen host interactions

**Figure 8:**
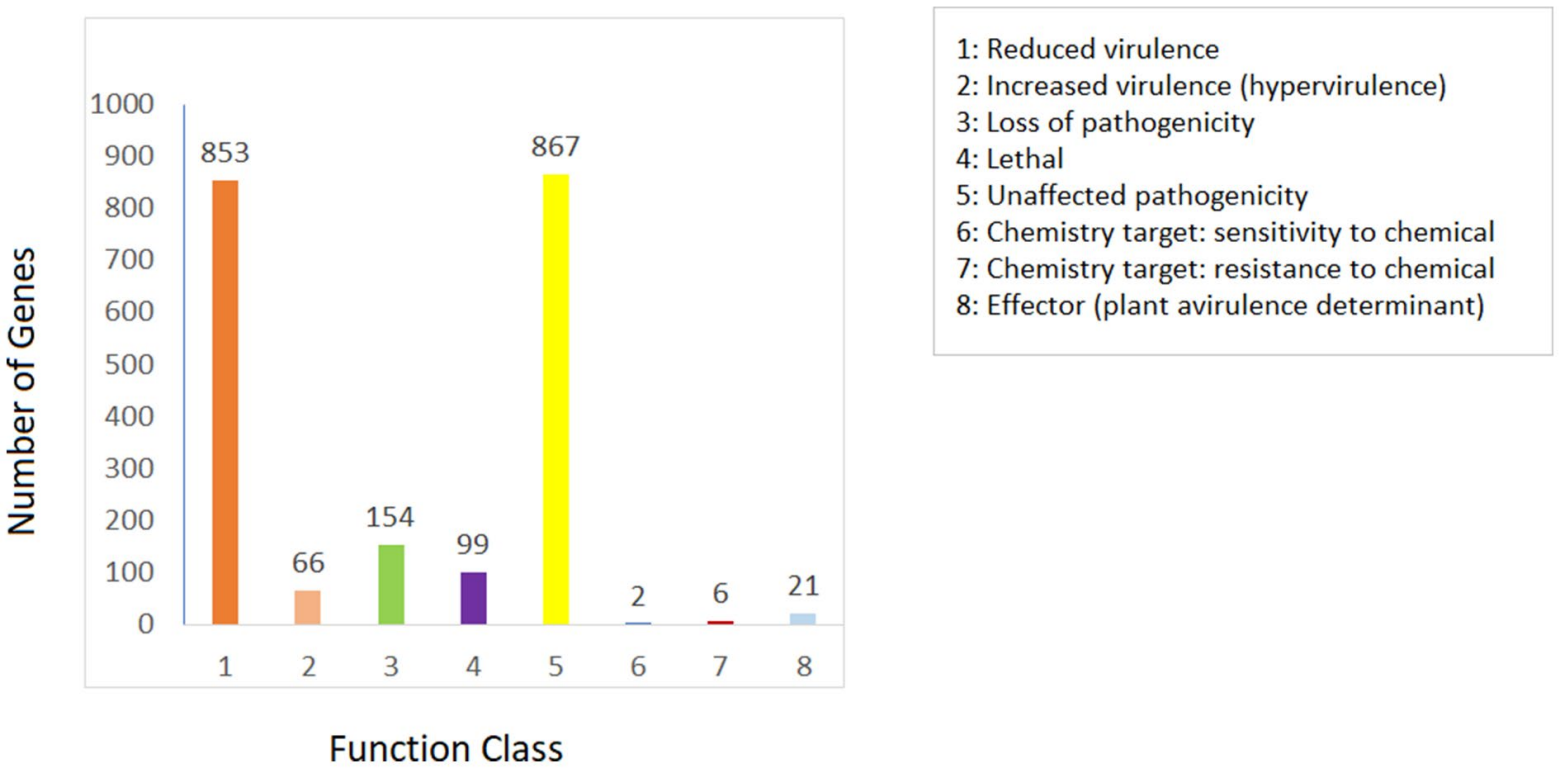
Distribution map of mutation types in the pathogen PHI phenotype of *Fusarium indicum* NFCCI 5145.

The Pathogen Host Interactions Database (PHI-base) is a database that is manually curated by experts based on experimental evidence that consists of genes related to virulence, effector, and pathogenicity from fungi, bacteria, and oomycete that infect plants, animals, insects, and fungi (Urban et al., 2022). The amino acid sequence of *Fusarium indicum* NFCCI 5145 was compared with the PHI database. As shown in Figure 8, *Fusarium indicum* NFCCI 5145 possesses abundant PHI-base genes, including reduced virulence (853), increased virulence (hypervirulence; 66), loss of pathogenicity (154), lethal (99), unaffected pathogenicity (867), sensitivity to chemical (2), effector (plant avirulence determinant; 21), and resistance to chemical (6; Pernas, 2021). Unaffected pathogenicity and reduced virulence were the major annotation genes, indicating that *Fusarium indicum* NFCCI 5145 is not a pathogenic strain as expected as it was isolated as an endophyte.

### 3.7 AntiSMASH analysis of *Fusarium indicum*

**Figure 9:**
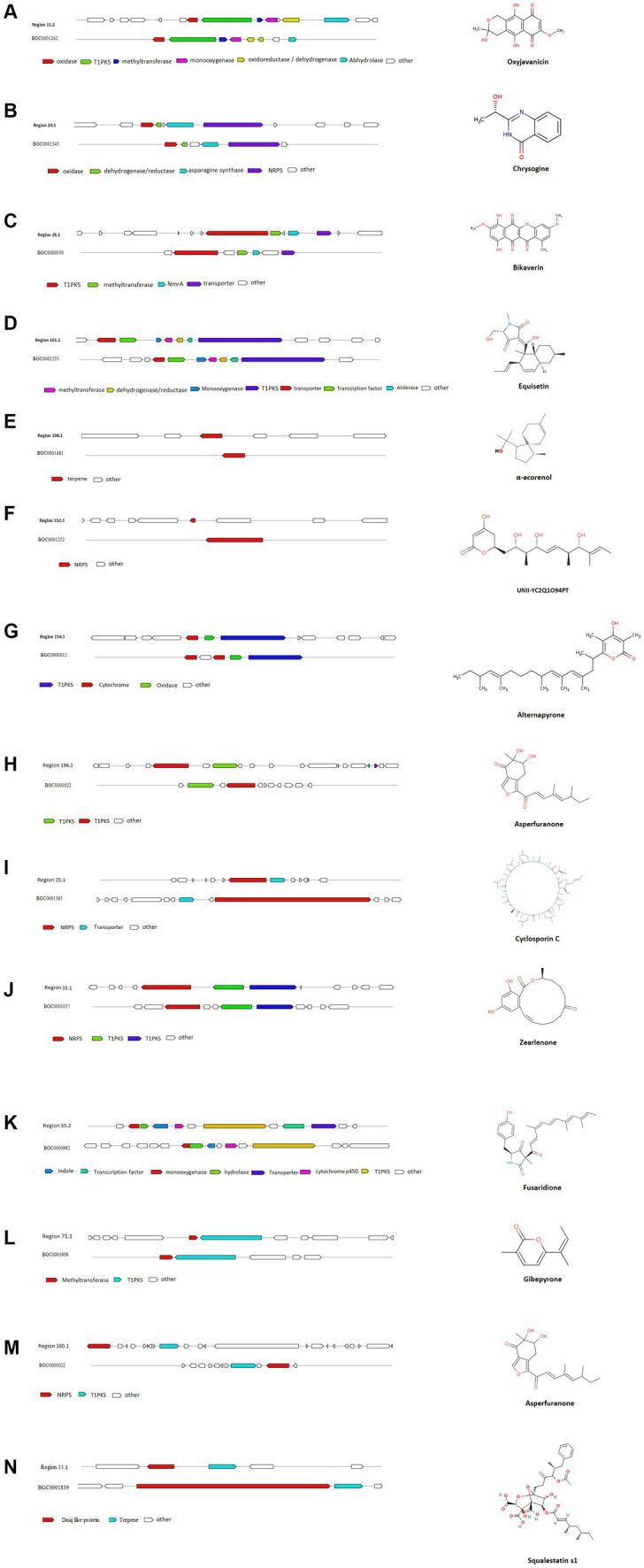
Comparison of biosynthetic gene cluster components in *Fusarium indicum* NFCCI 5145 with known biosynthetic gene clusters for biosynthesis of **(A)** Oxyjavanicin; **(B)** Chrysogine; **(C)** Bikaverin; **(D)** Equisetin; **(E)** α-Acorenol; **(F)** Unii-Yc2q1o94pt; **(G)** Alternapyrone; **(H)** Asperfuranone; **(I)** Cyclosporin C; **(J)** Zearalenone; **(K)** Fusaridione A; **(L)** Gibepyrone-A; **(M)** Asperfuranone; **(N)** Squalestatin S1.

AntiSMASH results indicated that *Fusarium indicum* isolate NFCCI 5145 contains 45 secondary metabolite biosynthetic gene clusters (BGCs), including eight NRPS-like fragments (NRPS-like), nine nonribosomal peptide synthetases (NRPSs), six terpene synthases, 12 Type I PKS (polyketide synthase; T1PKS), one Type III PKS (polyketide synthase; T3PKS), one beta-lactone containing protease inhibitor (betalactone), three hybrid NRPS + T1PKS, two indoles, one hybrid indole + NRPS, one hybrid T1PKS and NRPS, and one hybrid NRPS, T1PKS and indole (Table 3; Figure 9A–N).

**Table 3:** Putative biosynthetic gene clusters (BGCs) coding for secondary metabolites in strain *Fusarium indicum* NFCCI 5145.

Region	Type	From	To	Most similar known cluster (similarity %)
Region 4.1	NRPS-like	1	33,561	Unknown
Region 11.1	Terpene	573,526	595,242	Squalestatin S1 (40%)
Region 11.2	T1PKS	1,184,223	1,231,768	Oxyjavanicin (87%)
Region 24.1	NRPS	755,097	803,518	Chrysogine (66%)
Region 25.1	NRPS	1	48,656	Cyclosporin C (15%)
Region 26.1	T1PKS	6,912	55,582	Bikaverin (57%)
Region 30.1	Terpene	243,731	264,969	Unknown
Region 33.1	NRPS, T1PKS	1	62,940	Zearalenone (27%)
Region 40.1	NRPS	133,541	196,740	Unknown
Region 45.1	NRPS-like	531,411	571,016	Unknown
Region 45.2	NRPS, T1PKS	854,727	907,199	Unknown
Region 45.3	Indole	909,834	931,151	Unknown
Region 55.1	T1PKS	20,999	61,587	Unknown
Region 55.2	NRPS, T1PKS, Indole	63,907	116,020	Fusaridione A (31%)
Region 62.1	NRPS	619,119	660,567	Unknown
Region 66.1	NRPS-like	565,708	609,661	Unknown
Region 66.2	T1PKS	756,957	805,095	Unknown
Region 71.1	T1PKS	33,621	82,104	Gibepyrone-A (40%)
Region 82.1	NRPS-like	57,931	102,954	Unknown
Region 100.1	T1PKS, NRPS	359	110,394	Asperfuranone (18%)
Region 101.1	NRPS, T1PKS	21,869	74,188	Equisetin (63%)
Region 101.2	T1PKS	126,661	187,340	Unknown
Region 108.1	Terpene	261,099	282,316	α-Acorenol (100%)
Region 124.1	Betalactone	364,899	398,051	Unknown
Region 127.1	NRPS-like	21,088	64,945	Unknown
Region 138.1	Terpene	1	11,171	Unknown
Region 152.1	NRPS-like	43,150	81,103	Unknown
Region 152.2	NRPS-like	127,402	170,482	Unknown
Region 152.3	NRPS	194,208	239,103	Unii-Yc2q1o94pt (100%)
Region 154.1	T1PKS	1	48,664	Alternapyrone (80%)
Region 161.1	T3PKS	292,604	334,083	Unknown
Region 161.2	T1PKS	649,946	703,060	Unknown
Region 161.3	T1PKS	1,080,589	1,128,494	Unknown
Region 165.1	Indole	42,388	55,659	Unknown
Region 166.1	NRPS-like	1,329,329	1,373,093	Unknown
Region 170.1	NRPS, Indole	438,798	502,870	Unknown
Region 171.1	T1PKS	10,299	58,834	Unknown
Region 183.1	NRPS	84,248	141,704	Unknown
Region 183.2	Terpene	350,987	372,928	Unknown
Region 196.1	T1PKS	33,613	129,483	Asperfuranone (18%)
Region 201.1	T1PKS	379,627	427,143	Unknown
Region 206.1	NRPS	695,719	743,260	Unknown
Region 211.1	NRPS	193,558	239,017	Unknown
Region 211.2	NRPS	398,810	455,174	Unknown
Region 229.1	Terpene	40,056	53,407	Unknown

AntiSMASH results revealed the potential of this isolate to produce interesting compounds, such as α-acorenol, oxyjavanicin, chrysogine, equisetin, bikaverin, squalestatin S1, and many other known and unknown secondary metabolites. α-Acorenol is a highly oxygenated sesquiterpene that has antioxidant activity (Elshamy et al., 2019). Oxyjavanicin belongs to the naphthoquinone class of compounds and is similar to Fusarubin (C_15_H_14_O_7_), a red antibiotic (Ruelius and Gauhe, 1950). Many studies have reported its antituberculosis, cytotoxic, and antimicrobial activities; it can be a beneficial drug for the treatment of asthma as it regulates cytokine balance in OVA-sensitized (Hong et al., 2022). Fusarubin is also known to inhibit proliferation and also increase apoptosis in cell lines derived from hematological cancers (Adorisio et al., 2019). Chrysogine is a yellow pigment known to lack antimicrobial or anticancer activity (Viggiano et al., 2018). Equisetin is more known for its antibiotic and cytotoxic activity; it also inhibits HIV-1 integrase. It can also potentiate antibiotic activity against multi-drug-resistant gram-negative bacteria (Zhang et al., 2021). It is known to inhibit bacterial acetyl-CoA carboxylase (ACC), which is the first step of fatty acid synthesis (Larson et al., 2020). Bikaverin belongs to the polyketide class of compounds and is a reddish pigment having many biological properties including antitumoral activity against different cancer cell lines (Limón et al., 2010). Squalestatin S1 is a potent inhibitor of squalene synthase with potential use in the control of cholesterol biosynthesis (Lebe and Cox, 2018). The functions associated with other cryptic BGCs can be characterized with the help of gene knockout and heterogeneous expression experiments, along with the help of LC-MS analysis.

### 3.8 Comparative phylogenetics and genomics

#### 3.8.1 The average nucleotide identity

**Figure 10:**
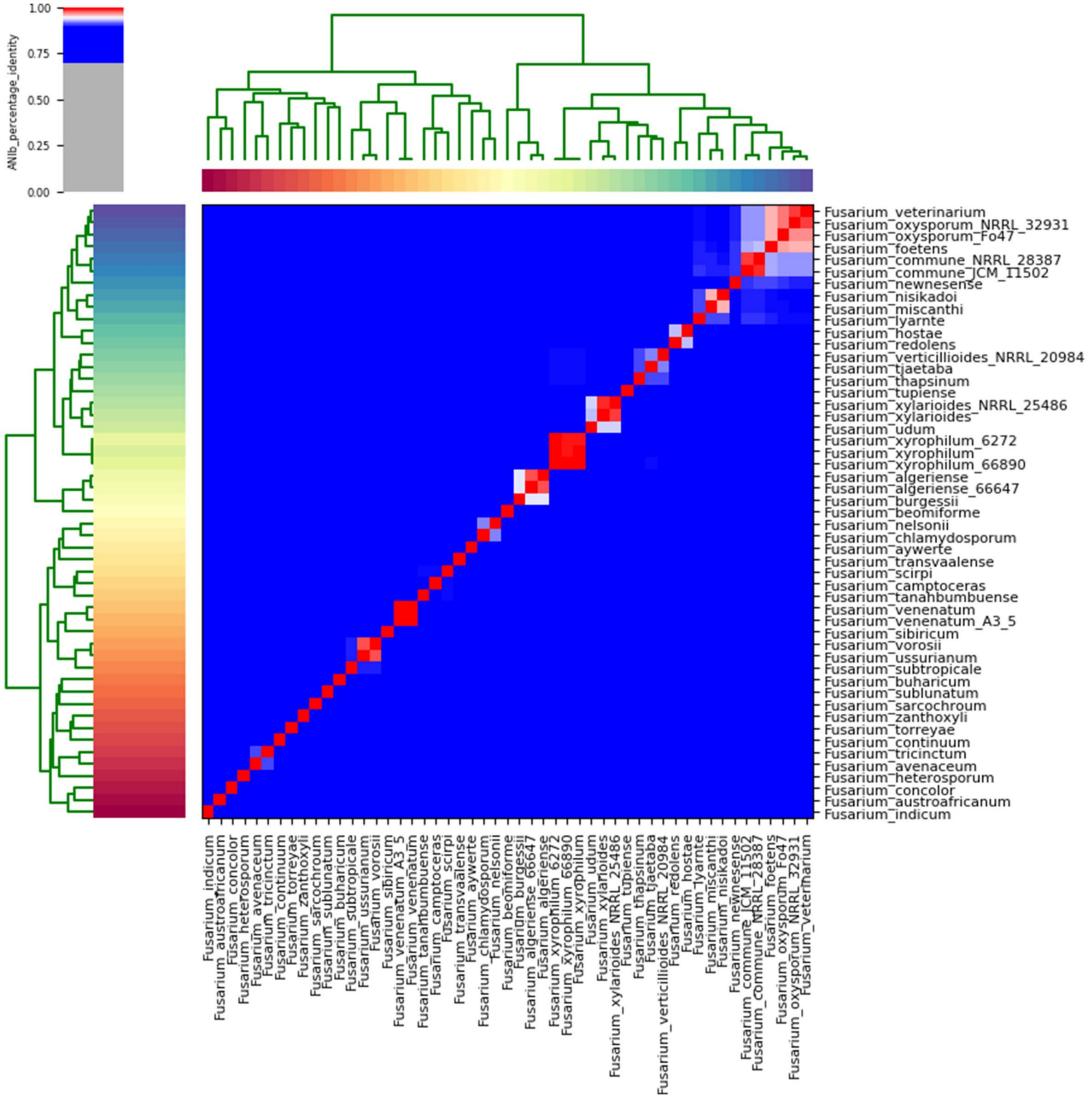
Heatmap of ANIb percentage identity between the *Fusarium* strains under comparison with the *Fusarium indicum* strain NFCCI 5145. ANIb was for all 51 genomes calculated based on genome sequences.

The average nucleotide identity (ANI) performed on the *Fusarium indicum* NFCCI 5145 genome provided an overall idea of the sequence identity between the *Fusarium* strains under comparison with the *F. indicum* strain NFCCI 5145 as depicted in the heatmap in Figure 10. *Fusarium indicum* NFCCI 5145 was grouped with *F. concolor* and *F. austroafricanum*, thus confirming the clustering of our strain *Fusarium indicum* NFCCI 5145 within the *Fusarium concolor* species complex. ANIb analysis included 51 genomes of various species of *Fusarium*.

#### 3.8.2 Phylogeny based on orthologous proteins

**Figure 11:**
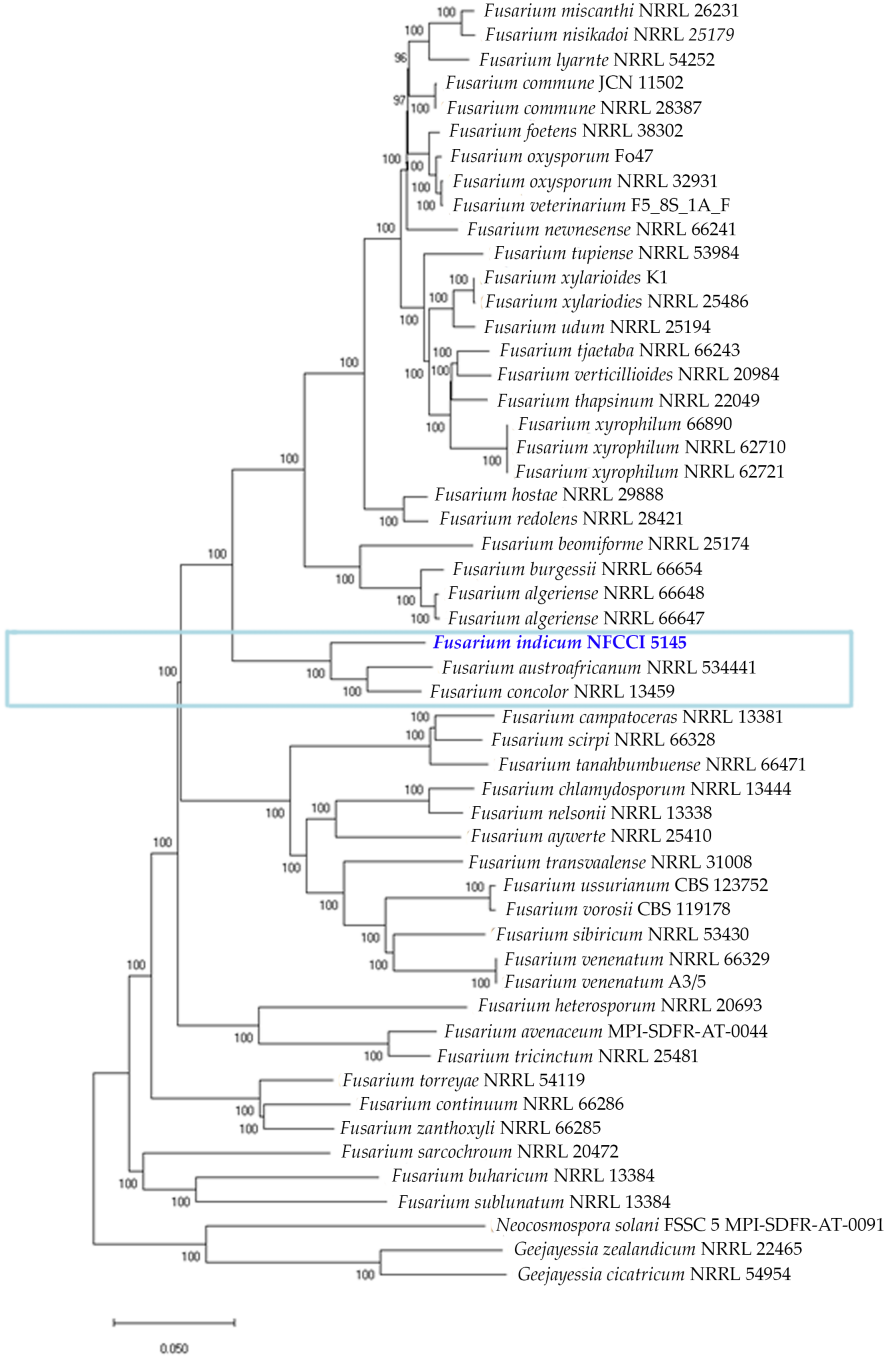
Phylogenetic analysis of 53 *Fusarium* strains based on the orthologous proteins identified using OrthoFinder. The new species is represented with blue bold and the *Fusarium concolor* species complex is marked with a blue rectangular box.

*Fusarium indicum* strain NFCCI 5145 was grouped in the *Fusarium concolor* species complex, which presently contains two species, *F. concolor* and *F. austroafricanum.* This was based on the phylogenetic tree constructed using the orthologous proteins of *Fusarium* strains, figured out using orthofinder. This confirms the clustering of the strain NFCCI 5145 in the *Fusarium concolor* species complex (Figure 11).

## 4 Conclusion

Species of *Fusarium* produce a variety of secondary metabolites. In this study, a novel endophytic, *Fusarium indicum*, isolated from *Bambusa* sp., was obtained and comprehensively examined by gene prediction and annotation. This isolate has many functional genes for energy production and conversion, amino acid and carbohydrate metabolism, secondary metabolites biosynthesis, transport, and catabolism. AntiSMASH analysis showed that it could produce secondary metabolites for drug development. According to the reports, species belonging to *Fusarium concolor* can produce ligninolytic enzymes, such as lignin peroxidase, laccase, and manganese peroxidase on wheat straw, thus leading to efficient delignification under solid-state fermentation conditions (Li et al., 2008). Species falling under this complex have also been known to treat poplar chemi-thermomechanical pulp to inhibit light-induced yellowing and enhance the brightness of the pulp (Daolei et al., 2020). Reports also establish the usage of an endophytic *Fusarium concolor* in synthesizing silver nanoparticles (Almeida et al., 2021). This newly isolated strain may be used for similar applications, such as the pretreatment of lignocellulose materials before bio-pulping or the bioconversion to fuel (Li et al., 2008). Nowadays, nanoparticles are used for the controlled release of pesticides or nanocides and the production of nanofertilizers. This isolate can be used to produce nanoparticles that may have great applicability in agriculture, biotechnology, and medicine.

## Data availability statement

The datasets presented in this study can be found in online repositories. The names of the repository/repositories and accession number(s) can be found in the article/Supplementary material.

## Author contributions

SR: Conceptualization, Data curation, Formal analysis, Investigation, Methodology, Software, Validation, Visualization, Writing – original draft. SS: Conceptualization, Data curation, Formal analysis, Investigation, Methodology, Project administration, Software, Supervision, Validation, Visualization, Writing – review & editing.

## Footnotes

^1^https://github.com/OpenGene/fastp+

^2^http://cab.spbu.ru/software/spades/

^3^http://bioinf.uni-greifswald.de/webaugustus/

^4^https://tandem.bu.edu/trf/trf.html

^5^https://blast.ncbi.nlm.nih.gov/Blast.cgi?PROGRAM=blastn on date 18.07.2023
