# Supplementary material for: Insights into the genomic architecture of a newly discovered endophytic Fusarium species belonging to the Fusarium concolor complex from India
Source: Front Microbiol. 2023 Nov 27;14:1266620. doi: 10.3389/fmicb.2023.1266620 (PMC10712836; doi:10.3389/fmicb.2023.1266620)
Supplement: Supplementary file 1 [file Table_1.docx]

Supplementary Material

Insights into the Genomic Architecture of a Newly Discovered Endophytic *Fusarium* Species belonging to the *Fusarium concolor* Complex from India

Shiwali Rana, Sanjay K. Singh*

*** Correspondence:** Corresponding Author: [sksingh@aripune.org](mailto:sksingh@aripune.org)

**Supplementary Table 1.** Details of the selected strains used in the construction of the phylogenetic tree of novel *Fusarium indicum* NFCCI 5145.

| **Sr. no.** | ***Fusarium* spp.** | **Isolate** | **Locality** | **Substrate** | ***rpb2*** | ***tef-1α*** |
| --- | --- | --- | --- | --- | --- | --- |
| 1 | *Fusarium algeriense* | CBS 142638 = NRRL 66647 | Algeria, Guelma Province, Djeballah Khemissi | *Triticum durum* | MF120499 | MF120510 |
| 2 | *F.* *anguioides* | LC13612 | China, Guangdong Province, Shenzhen city | *Cordyline stricta* | MW474381 | MW580435 |
| 3 | *F. atrovinosum* | CBS 445.67 = BBA 10357 = DSM 62169 = IMI 096270 = NRRL 26852 = NRRL 26913 | Australia | *Triticum aestivum* | MW928822 | MN120752 |
| 4 | *F. austroafricanum* | NRRL 66741 = PPRI 10408 | South Africa, Eastern Cape Province, Humansdorp | Endophyte of *Pennisetum clandestinum* | MH742616 | MH742687 |
| 5 | *F. avenaceum* | CBS 408.86 = FRC R-8510 = IMI 309354 = NRRL 26850 = NRRL 26911 | Denmark | *Hordeum*  *vulgare* | MG282401 | MW928836 |
| 6 | *F. aywerte* | F10108 = NRRL 25410 | Australia, Northern Territory, Deep Well | Soil | JX171626 | JABCQV010000336 |
| 7 | *F. bambusarum* |  | China, Jiangxi Province | Bamboo | MW474389 | MW580443 |
| 8 | *F. bambusarum* |  | China, Guangdong Province, Guangzhou city | Bamboo | MW474390 | MW580444 |
| 9 | [*F. beomiforme*](https://www.fusarium.org/page/TaxonomyDisplay/39) | CBS 100160 = DAR 58880 = FRC M-1425 = IMI 316127 = MRC 4593 = NRRL 13606 | Australia, Queensland, Rockhampton | Soil | MF120496 | MF120507 |
| 10 | [*F. buharicum*](https://www.fusarium.org/page/TaxonomyDisplay/46) | CBS 178.35 = DSM 62166 = NRRL 25488 | Uzbekistan, Tashken | *Gossypium herbaceum* | KX302928 | KX302912 |
| 11 | [*F. burgessii*](https://www.fusarium.org/page/TaxonomyDisplay/48) | CBS 125537 = NRRL 66654 = RBG 5315 | Australia, Queensland, Idalia National Park | Soil | HQ646393 | HQ667148 |
| 12 | *F. camptoceras* | ATCC 16065 = ATCC 24364 = BBA 9810 = CBS 193.65 = DSM 62167 = IMI 112500 = NRRL 20716 = NRRL 36344 | Costa Rica | Cushion gall of *Theobroma cacao* | MN170383 | MN170450 |
| 13 | [*F. cassiae*](https://www.fusarium.org/page/TaxonomyDisplay/54) | MFLUCC 18-0573 | Thailand, Phayao Province | Pods of *Cassia fistula* | MT212197 | MT212205 |
| 14 | *F. chlamydosporum* | CBS 145.25 = NRRL 26851 = NRRL 26912 | Honduras, Tela | *Musa sapientum* | MN120735 | MN120754 |
| 15 | *F. citricola* | CBS 142421 = CPC 27805 | Italy, Cosenza, Rocca Imperiale | *Citrus reticulata* ‘Caffin’ | LT746310 | LT746197 |
| 16 | *F. commune* | AAS 156 = BBA 71639 = CBS 110090 = NRRL 31076 | Denmark | Soil | MW934368 | AF362263 |
| 17 | *F. concolor* | BBA 2607 = BBA 63601 = CBS 183.34 = DAOM 225131 = DSM 62179 = IMI 112502 = NRRL 13994 | Uruguay, Montevideo | *Hordeum vulgare* | MH742569 | MH742650 |
| 18 | *F. continuum* | CBS 140841 = F201030 = NRRL 66286 | China, Shaanxi, Fuping, Lei village | *Zanthoxylum bungeanum* | KM236782 | KM236722 |
| 19 | [*F. convolutans*](https://www.fusarium.org/page/TaxonomyDisplay/73) | CBS 144207 = CPC 33733 | South Africa, Kruger National Park, Skukuza, Granite Supersite | Rhizosphere of *Kyphocarpa angustifolia* | LT996141 | LT996094 |
| 20 | *F. foetens* | CBS 110286 = NRRL 31852 = PD 2001/7244 | Netherlands, Zuid-Holland Province, Maasland | *Begonia elatior* hybrid | MW928825 | AY320087 |
| 21 | *F. gamsii* | CBS 143610 = CPC 30862 | Iran, West Azerbaijan Province, Orumieh-Salmas | *Agaricus bisporus* | LT970760 | LT970788 |
| 22 | *F. glycines* | CBS 144746 = CPC 25808 | South Africa, North West Province | *Glycine max* | MH484942 | MH485033 |
| 23 | *F. gossypinum* | CBS 116613 | Ivory Coast | *Gossypium hirsutum* | MH484909 | MH485000 |
| 24 | *F. grosmichelii* | InaCC F833 | Indonesia, West Java, Bogor, Suakarya  (Megamendung) | Pseudostem of *Musa acuminata* | LS479295 | LS479744 |
| 25 | *F. heterosporum* | CBS 391.68 = NRRL 25798 | Germany, Rotenburg near Bremen | Sclerotium of *Claviceps* *purpurea* on *Lolium perenne* | MW928827 | MW928839 |
| 26 | *F. hostae* | FRC O-2074 = NRRL 29889 | USA, South Carolina | *Hosta* sp. | JX171640 | AY329034 |
| 27 | *F. humicola* | ATCC 24372 = CBS 124.73 = IMI 128101 = NRRL 25535 | Pakistan | Soil | MN120738 | MN120757 |
| **28** | ***F. indicum**** | **NFCCI 5145** | **India, Himachal Pradesh, Panchrukhi** | **Leaves of *Bambusa* sp.** | **OM032812** | **OM032811** |
| 29 | *F. iranicum* | CBS 143608 = CPC 30860 | Iran, West Azerbaijan Province, Orumieh-Salmas | *Agaricus bisporus* | LT970757 | LT970785 |
| 30 | *F. kotabaruense* | InaCC F963 | Indonesia, South Kalimantan, Kota Baru, Kecamatan Pamukan Barat, Desa Sungai Birah | *Musa* var. Pisang Hawa | LS479859 | LS479445 |
| 31 | *F. libertatis* | CBS 144749 = CPC 28465 | South Africa, Western Cape Province, Robben  Island, Van Riebeeck's Quarry | Rock surface | MH484944 | MH485035 |
| 32 | *F. lyarnte* | CBS 125536 = NRRL 54252 = RBG 5331 | Australia, Northern Territory, Litchfield | Soil | JX171661 | EF107118 |
| 33 | [*F. massalimae*](https://www.fusarium.org/page/TaxonomyDisplay/146) | URM 8239 | Brazil, Alagoas, Quebrangulo, Pedra Talhada  Biological Reserve | *Handroanthus chrysotrichus* | MN939767 | MN939763 |
| 34 | [*F. microconidium*](https://www.fusarium.org/page/TaxonomyDisplay/150) | CBS 119843 = MRC 8391 = KSU 11396 | Unknown | Unknown |  | MN120759 |
| 35 | *F. miscanthi* | CBS 577.97 = NRRL 26231 | Denmark, Zealand, Højbakkegård Experimental field | *Miscanthus sinensis* | JX171634 | MN193878 |
| 36 | [*F. nelsonii*](https://www.fusarium.org/page/TaxonomyDisplay/160) | CBS 119876 = FRC R-8670 = MRC 4570 = NRRL 28505 = NRRL 53945 | South Africa, Western Cape Province, Malmesbury | Plant debris in *Triticum* soil | GQ505468 | GQ505404 |
| 37 | *F. neoscirpi* | CBS 610.95 = NRRL 26861 = NRRL 26922 | France | Soil | GQ505779 | GQ505601 |
| 38 | *F. neosemitectum* | CBS 189.60 | Democratic Republic of the Congo | *Musa sapientum* | MN170422 | MN170489 |
| 39 | *F. newnesense* | NRRL 66241 = RBG 610 | Australia, New South Wales, Newnes State Forest | Soil | JABCJW010000963 | KP083261 |
| 40 | *F. nirenbergiae* | CBS 840.88 | Netherlands, Noord-Holland Province, Aalsmeer | *Dianthus caryophyllus* | MH484887 | MH484978 |
| 41 | *F. nisikadoi* | BBA 69015 = CBS 456.97 = MAFF 237506 = NRRL 25205 = NRRL 25308 | Japan, Oita, Hita | *Triticum aestivum* | MG282421 | KR909358 |
| 42 | *F. nurragi* | CBS 393.96 = DAR 69501 = F10108 = F11121 | Australia, Victoria, Wilson's PromontoryNational Park | Soil | MW928830 | MW928840 |
| 43 | *F. oxysporum* | CBS 144134 | Germany, Berlin | *Solanum tuberosum* | MH484953 | MH485044 |
| 44 | *F. pernambucanum* | MUM 1862 = URM 7559 | Brazil, Pernambuco, Paudalho | *Aleurocanthus woglumi* | LS398519 | LS398489 |
| 45 | *F. persicinum* | CBS 479.83 | Unknown | Unknown | MN170428 | MN170495 |
| 46 | [*F. peruvianum*](https://www.fusarium.org/page/TaxonomyDisplay/177) | CBS 511.75 | Peru | Seedlings of *Gossypium* sp. | MN120746 | MN120767 |
| 47 | *F. petersiae* | CBS 143231 | Netherlands, Gelderland Province, Arnhem | Soil | MG386150 | MG386160 |
| 48 | *F. pharetrum* | CBS 144751 = CPC 30824 | South Africa | *Aloidendron dichotomum* | MH484952 | MH485043 |
| 49 | *F. pseudograminearum* | FRC R-5291 = NRRL 28062 | Australia, New South Wales, Young | *Hordeum vulgare* | JX171637 | AF212468 |
| 50 | *F. redolens* | ATCC 16067 = BBA 9526 = CBS 248.61 = CBS 360.87 = DSM 62390 = NRRL 20426 = NRRL 25600 | Germany, Berlin-  Dahlem | Vascular bundle of *Dianthus caryophyllus* | MT409443 | MT409453 |
| 51 | *F. robustum* | BBA 63667 = CBS 637.76 = FRC R-5821 = IMI 322102 = NRRL 13392 | Argentina | *Araucaria angustifolia* | MW928831 | MW928842 |
| 52 | *F. salinense* | CBS 142420 = CPC 26973 | Italy, Sicily, Messina, Len | Twigs of *Citrus sinensis* | LT746306 | LT746193 |
| 53 | *F. sarcochroum* | BBA 63714 = CBS 745.79 = NRRL 20472 | Switzerland | *Viscum album* | JX171586 | MW834278 |
| 54 | *F. scirpi* | CBS 447.84 = FRC R-6252 = NRRL 36478 | Australia, New  South Wales, near Broken Hill | Pasture soil | GQ505832 | GQ505654 |
| 55 | *F. serpentinum* | BBA 62209 = CBS 119880 = MRC 1813 | Unknown | Unknown | MN170432 | MN170499 |
| 56 | *F. sibiricum* | MFG 11013 = NRRL 53430 | Russia, Khabarovsk | Grain of *Avena sativa* | HQ154472 | HM744684 |
| 57 | *F. spartum* | NRRL 66896 | Tunisia, Kasserine Governorate | Rhizosphere of *Macrochloa tenacissima* | MT409449 | MT409459 |
| 58 | [*F. spinosum*](https://www.fusarium.org/page/TaxonomyDisplay/208) | CBS 122438 | Brazil | *Galia melon* imported into the Netherlands | MN120747 | MN120768 |
| 59 | *F. sporodochiale* | ATCC 14167 = CBS 220.61 = MUCL 8047 = NRRL 20842 | South Africa, Gauteng Province, Johannesburg | Soil | MN120749 | MN120770 |
| 60 | *F. stilboides* | BBA 63887 = CBS 746.79 = ICMP 10624 = NRRL 25485 | Cook Islands | *Citrus*  sp. | MW928832 | MW928843 |
| 61 | [*F. sublunatum*](https://www.fusarium.org/page/TaxonomyDisplay/214) | BBA 62431 = CBS 189.34 = DSM 62431 = NRRL 13384 = NRRL 20840 | Costa Rica, Limon | Soil from *Musa sapientum* plantation | KX302935 | KX302919 |
| 62 | *F. subtropicale* | CBS 144706 = NRRL 66764 | Brazil, Parana State, Guarapuava | *Hordeum vulgare* | MH706973 | MH706974 |
| 63 | *F. sulawesiense* | InaCC F940 | Indonesia, South Sulawesi, Bone, Kecamatan  Bengo, Desa Selli | Infected Pseudostem of *Musa acuminata* var.  Pisang Cere (AAA) | LS479855 | LS479443 |
| 64 | *F. tanahbumbuense* | InaCC F965 | Indonesia, South Kalimantan, Tanah Bumbu,  Kecamatan Kusan Hilir, Desa Betung | Pseudostem of *Musa* var. Pisang Hawa | LS479863 | LS479448 |
| 65 | *F. terricola* | CBS 483.94 | Australia, Queensland | Desert soil | LT996156 | KU711698 |
| 66 | [*F. thapsinum*](https://www.fusarium.org/page/TaxonomyDisplay/224) | ATCC 200522 = CBS 777.96 = FRC M-6564 | USA, Kansas | Stalk of *Sorghum* sp. | MW928833 | MW928844 |
| 67 | *F. tjaetaba* | FRL14350 = NRRL 66243 = RBG 5361 | Australia, Northern Territory, Litchfield National Park | *Sorghum interjectum* | KP083275 | KP083263 |
| 68 | *F. tjaynera* | NRRL 66246 = RBG 5367 | Australia, Northern Territory, Litchfield National Park | *Triodia microstachya* | KP083279 | EF107152 |
| 69 | *F. torreyae* | CBS 133858 = MAFF 243468 = NRRL 54151 | USA, Florida, Liberty County, Torreya State Park,  Aspalaga Tract | Stem tissue of diseased *Torreya taxifolia* | MW928834 | MW928845 |
| 70 | *F. toxicum* | CBS 406.86 = FRC R-8507 = IMI 309347 = NRRL 25796 | Germany, Berlin | Soil | MN170441 | MN170508 |
| 71 | *F. transvaalense* | CBS 144211 | South Africa, Kruger National Park, Skukuza, Granite Supersite | Rhizosphere of *Sida cordifolia* | LT996157 | LT996099 |
| 72 | *F. tricinctum* | BBA 64485 = CBS 393.93 = NRRL 25481 | Germany, Berlin | Culm base of *Triticum aestivum* | JX171629 | MH582379 |
| 73 | [*F. tupiense*](https://www.fusarium.org/page/TaxonomyDisplay/232) | CML 262 = CMM 3655 = KSU 16195 = NRRL 53984 | Brazil, Minas Gerais, Lavras | Diseased tissue of *Mangifera indica* | LR792619 | GU737404 |
| 74 | [*F. udum*](https://www.fusarium.org/page/TaxonomyDisplay/233) | BBA 65058 = CML 3238 = NRRL 25199 | India | *Cajanus cajan* | KY498875 | MK639096 |
| 75 | *F. ussurianum* | CBS 123752 = NRRL 45681 = TG-2662/0 | Russia, Ussuriysk, Primorsky krai (Far East territory), agricultural field near the city Ussuriysk | Seed of *Avena sativa* | KM361666 | FJ240301 |
| 76 | *F. venenatum* | BBA 64537 = CBS 458.93 = NRRL 26228 | Austria | Culm of *Triticum aestivum* | KM232382 | KM231942 |
| 77 | *F. verticillioides* | BBA 11782 = CBS 218.76 = DSM 62264 = IMI 202875 = NRRL 13993 | Germany | *Zea mays* | MW928835 | KF499582 |
| 78 | *F. veterinarium* | CBS 109898 = NRRL 36153 | Netherlands | Peritoneum of *Selachimorpha* | MH484899 | MH484990 |
| 79 | [*F. volatile*](https://www.fusarium.org/page/TaxonomyDisplay/238) | CBS 143874 | French Guiana, Cayenne | Bronchoalveolar lavage effusion from *Homo* *sapiens* | LR596006 | LR596007 |
| 80 | *F. vorosii* | NRRL 37605 | Hungary, Pest, Ipolydamasd | Spikelet of *Triticum aestivum* | KM361665 | DQ459745 |
| 81 | *F. werrikimbe* | CBS 125535 = F19350 = RBG 5332 | Australia, New South Wales, Werrikimbe National Park | *Sorghum leiocladum* | MN534304 | MW928846 |
| 82 | [*F. xylarioides*](https://www.fusarium.org/page/TaxonomyDisplay/241) | CBS 258.52 = NRRL 25486 | Ivory Coast | Trunk of *Coffea* sp. | JX171630 | AY707136 |
| 83 | [*F. xyrophilum*](https://www.fusarium.org/page/TaxonomyDisplay/242) | FRC M-8921 = NRRL 62721 | Guyana, Cuyuni-Mazaruni, Kamakusa Mountain | *Xyris surinamensis* | MN193905 | MN193877 |
| 84 | *F. zanthoxyli* | CBS 140838 = NRRL 66285 | China, Shaanxi, Tongchuan, Yaozhou, Sunyuan | *Zanthoxylum bungeanum* | KM236763 | KM236703 |
| 85 | *Geejayessia cicatricum* | CBS 125549 | Slovenia, Arboretum Volcji Potok | Decaying twigs of *Buxus sempervirens* | HM626679 | HM626643 |
| 86 | *G. zealandica* | BBA 64792 = CBS 111.93 | New Zealand, Auckland, Waitakere Ranges Regional Park, Cascades Kauri | Bark of *Hoheria populnea* | HM626684 | HQ728148 |

*Details of the new species reported are provided in bold.
